# O-GlcNAc forces an α-synuclein amyloid strain with notably diminished seeding and pathology

**DOI:** 10.1038/s41589-024-01551-2

**Published:** 2024-02-12

**Authors:** Aaron T. Balana, Anne-Laure Mahul-Mellier, Binh A. Nguyen, Mian Horvath, Afraah Javed, Eldon R. Hard, Yllza Jasiqi, Preeti Singh, Shumaila Afrin, Rose Pedretti, Virender Singh, Virginia M.-Y. Lee, Kelvin C. Luk, Lorena Saelices, Hilal A. Lashuel, Matthew R. Pratt

**Affiliations:** 1 Department of Chemistry, University of Southern California, Los Angeles, CA USA; 2 Laboratory of Molecular and Chemical Biology of Neurodegeneration, Institute of Bioengineering, School of Life Sciences, École Polytechnique Fédérale de Lausanne, Lausanne, Switzerland; 3 Center for Alzheimer’s and Neurodegenerative Diseases, Department of Biophysics, Peter O’Donnell Jr. Brain Institute, UT Southwestern Medical Center, Dallas, TX USA; 4 The Department of Pathology and Laboratory Medicine, Institute on Aging and Center for Neurodegenerative Disease Research, the Perelman School of Medicine, University of Pennsylvania, Philadelphia, PA USA; 5 Department Biological Sciences, University of Southern California, Los Angeles, CA USA

**Keywords:** Glycobiology, Protein aggregation, Post-translational modifications, Proteins

## Abstract

Amyloid-forming proteins such α-synuclein and tau, which are implicated in Alzheimer’s and Parkinson’s disease, can form different fibril structures or strains with distinct toxic properties, seeding activities and pathology. Understanding the determinants contributing to the formation of different amyloid features could open new avenues for developing disease-specific diagnostics and therapies. Here we report that O-GlcNAc modification of α-synuclein monomers results in the formation of amyloid fibril with distinct core structure, as revealed by cryogenic electron microscopy, and diminished seeding activity in seeding-based neuronal and rodent models of Parkinson’s disease. Although the mechanisms underpinning the seeding neutralization activity of the O-GlcNAc-modified fibrils remain unclear, our in vitro mechanistic studies indicate that heat shock proteins interactions with O-GlcNAc fibril inhibit their seeding activity, suggesting that the O-GlcNAc modification may alter the interactome of the α-synuclein fibrils in ways that lead to reduce seeding activity in vivo. Our results show that posttranslational modifications, such as O-GlcNAc modification, of α-synuclein are key determinants of α-synuclein amyloid strains and pathogenicity.

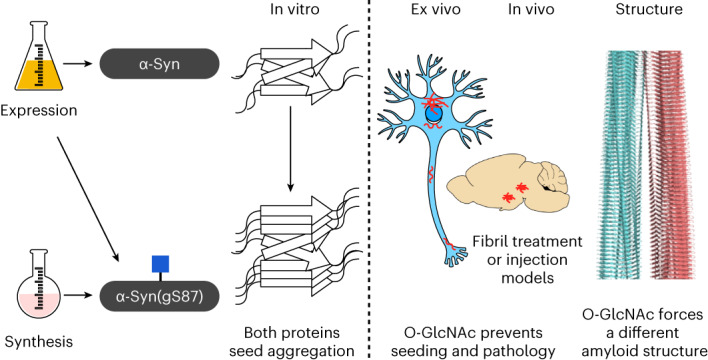

## Abstract (web summary)

α-Synuclein and tau can form multiple amyloid structures or strains that are associated with different neurodegenerative diseases, suggesting a strain–toxicity relationship. Now, it has been shown that O-GlcNAc modification of α-synuclein results in the formation of an amyloid strain that is largely nonpathogenic in vivo, supporting structure-dependent toxicity and another protective role for O-GlcNAc.

## Main

The formation and deposition of misfolded protein aggregates in the brain is a common feature of most neurodegenerative diseases (NDDs)^1,2^. Peptides and proteins that are entirely or partially unfolded in solution are prone to misfolding and formation of β-sheet-rich fibrillar aggregates characterized by the eventual stacking of monomers into β-sheets in a cross-β conformation, also known as amyloid fibrils^3^. The formation of these aggregates is associated with cell dysfunction and death. Several forms of misfolded protein oligomers and mature fibrils of different morphology and structural properties are toxic to mammalian cells and neurons in culture^4^. Additionally, amyloid fibrils derived from several proteins implicated in the pathogenesis of NDDs efficiently seed and induce the aggregation of the corresponding endogenous protein when added to neurons^5–7^ and spread through cell-to-cell mechanisms to different brain regions^8–12^. These observations, combined with the strong genetic and neuropathological evidence linking the aggregation of these proteins to the pathogenesis of NDDs, continue to drive strong interest in targeting the formation, pathogenic properties or removal of these amyloid structures as potential therapeutic strategies for the treatment of NDDs.

The ability of these individual proteins to form multiple amyloid structures or ‘strains’ in different NDDs presents substantial challenges, as therapies and diagnostics have not accounted for the structural and biochemical diversity of the different strains^13–15^. For example, alpha-synuclein (α-syn) fibrils isolated from patients with Parkinson’s disease (PD), multiple system atrophy (MSA) or Lewy body dementia (LBD) exhibit distinct structures^16–18^, and injection of aggregates isolated from these patients results in distinct phenotypes and pathology patterns in mice^19^. These results have generated notable interest in uncovering structure–pathogenicity relationships between these strains and the molecular and structural determinants of their formation. Posttranslational modifications (PTMs) can have profound consequences on the structure, biochemistry and function of proteins in health and disease. Biochemical studies of pathological hallmarks of NDDs have shown that they accumulate misfolded Aβ, tau and α-syn aggregates that are subjected to different types of PTMs at multiple residues^20–24^. Despite this, several questions regarding the roles of PTMs in NDDs remain unanswered, including (1) which PTMs enhance or protect against protein aggregation and toxicity in NDDs; (2) how do PTMs influence the structural, biochemical and cellular properties of fibrils; and (3) do they play critical roles in regulating α-syn seeding and pathology spreading?

**Fig. 1:**
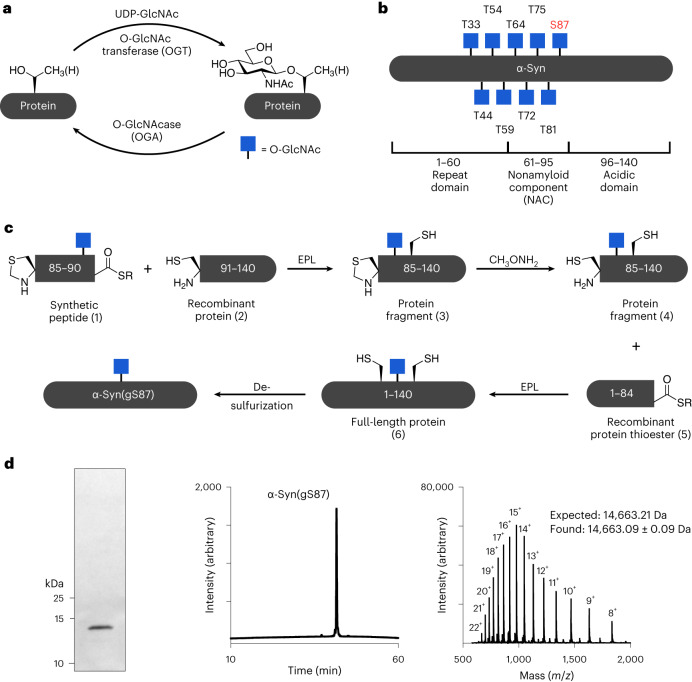
O-GlcNAc-modified α-syn. **a**, O-GlcNAc is the dynamic addition of *N*-acetylglucosamine to serine and threonine residues of intracellular proteins. **b**, α-Syn is O-GlcNAc modified in vivo at several residues that can alter its amyloid aggregation. The focus of this study, O-GlcNAc on serine 87 (gS87), is highlighted in red. **c**, Synthesis of α-syn(gS87) using expressed protein ligation. **d**, Characterization of α-syn(gS87) by gel electrophoresis, HPLC and mass spectrometry. Characterization of the protein was repeated for each batch of synthesis with no difference in the results. UDP, uridine diphosphate.

One such PTM, an intracellular form of glycosylation called O-GlcNAc (Fig. 1a)^25^, has been linked to several biological processes, including protein aggregation and neurodegeneration in several NDDs^26,27^. For example, O-GlcNAc levels are 40–50% lower in Alzheimer’s disease (AD) brains when compared with age-matched controls^28^, which is linked with tau hyperphosphorylation and neurodegeneration^29^. These and other observations have led to the creation of a range of O-GlcNAcase (OGA) inhibitors that can elevate O-GlcNAc modifications as potential therapeutics^30^. Multiple preclinical studies in animal models of AD and PD demonstrated that OGA inhibition increases O-GlcNAc in brains and slows the formation of amyloid aggregates and neuron death^31,32^. α-Syn is O-GlcNAc modified at multiple residues in vivo (Fig. 1b), and a recent analysis of α-syn in brain tissue from the Line 61 mouse model that overexpresses human α-syn brain found that 20% of the protein is O-GlcNAc modified and that these levels rise to ~35% upon OGA inhibition^32^. Some of these compounds have advanced to the clinic and show no overt toxicity in humans. We and others have shown that O-GlcNAc on tau and α-syn can directly slow the kinetics of amyloid aggregation of these proteins in vitro in a site-specific fashion^31,33–35^. These data indicate that O-GlcNAc may protect neurons by inhibiting amyloid aggregation and that increasing the levels of this modification with drugs may slow the progression of certain NDDs.

In our previous studies on α-syn O-GlcNAc modification^34,35^, we used synthetic protein chemistry to prepare the protein bearing O-GlcNAc at four different sites (T72, T75, T81 or S87). Biochemical analysis showed that individual O-GlcNAc residues slow the kinetics of α-syn aggregation with notable site-specific differences. However, these O-GlcNAc modifications did not completely stop the formation of α-syn aggregates over time. This led us to hypothesize that O-GlcNAc may alter the progression of PD by not only slowing the rate of amyloid formation but by causing the formation of amyloid strains with altered pathogenicity and toxicity.

In this Article, we applied protein synthesis in combination with biochemical, cellular, in vivo and structural analyses to test this hypothesis. We focused on O-GlcNAc modification at S87, termed α-syn(gS87), because this modification site displays the least inhibition of α-syn fibril formation, thus providing an opportunity to obtain homogeneously O-GlcNAc-modified fibrils for detailed analysis. We used a variety of in vitro experiments to show that α-syn(gS87) does form amyloid fibrils that have a different core structure and that these fibrils seed additional aggregation and template their structure onto the newly-formed α-syn aggregates. We observed that α-syn(gS87) fibrils failed to induce toxicity or α-syn aggregation in primary neurons. These results were recapitulated in vivo using seeding-mediate mouse models of α-syn aggregation and pathology spreading^7,12^. Mechanistic studies on α-syn seeding in neurons uncovered an interesting divergence in the behavior of the α-syn(gS87) fibrils, where they can seed aggregation in vitro but not in neurons or mice. We demonstrate that this may be due to differential protein–protein interactions including chaperones and used cryogenic electron microscopy (cryo-EM) to obtain a structural model of the α-syn(gS87) amyloid that is very different from both other in vitro fibrils and ex vivo aggregates from patients.

Our results confirm that O-GlcNAc can force the formation of a α-syn amyloid strain with diminished pathogenicity in neurons and in vivo. This adds evidence for a model where O-GlcNAc may not only slow the aggregation of α-syn, but could also protect against the progression of NDDs through multiple mechanisms. To our knowledge, this α-syn strain is also the only amyloid that is capable of strongly seeding aggregation in vitro but does not in neurons, and an example of a PTM that can almost completely block the seeding and spread of α-syn in vivo. More broadly, our results also demonstrate that some amyloids may be almost benign despite their ability to seed aggregation in vitro, with important implications for amyloid characterization and pathogenic structures in associated diseases.

## Results and discussion

### α-Syn(gS87) forms amyloid aggregates with a different structure

**Fig. 2:**
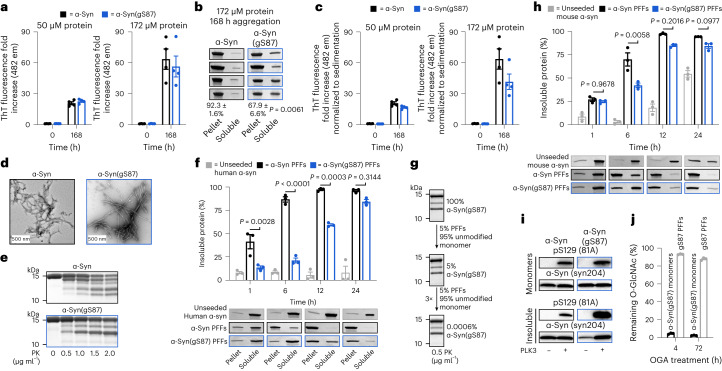
In vitro generation and characterization of α-syn(gS87) fibrils. **a**, α-Syn(gS87) aggregates into ThT-positive amyloids. α-Syn and α-syn(gS87) were subjected to aggregation conditions at the indicated concentrations before analysis by ThT fluorescence. The *y* axis shows a fold change in fluorescence compared with α-syn alone at *t* = 0 h. The results are mean ± s.e.m. of experimental replicates (*n* = 4). **b**, α-Syn(gS87) aggregates can be quantitated by sedimentation. α-Syn and α-syn(gS87) were subjected to aggregation conditions followed by sedimentation and visualization by Coomassie staining. The results are mean ± s.e.m. of experimental replicates (*n* = 4). Statistical significance was determined using a two-sided *t*-test without multiple comparisons. **c**, ThT data from **a** normalized to the sedimentation levels in **b**. **d**, The aggregation reactions in a (172 μM) were analyzed by TEM after 168 h. **e**, α-Syn(gS87) forms fibrillar aggregates of distinct structures as discerned from PK digestion. The aggregation reactions in **a** (172 μM) were subjected to the indicated concentrations of PK for 30 min before separation by SDS–PAGE and visualization by Coomassie staining. **f**, α-Syn(gS87) PFFs can seed aggregation of unmodified human α-syn. α-Syn PFFs (unmodified or gS87) were added to buffer or unmodified human α-syn (50 μM monomer concentration, 5% PFF) before aggregation and analysis by sedimentation and Coomassie staining. The results are mean ± s.e.m. of experimental replicates (*n* = 3). Statistical significance was determined using a one-way ANOVA test followed by Tukey’s multiple comparison test. **g**, α-Syn(gS87) amyloids template their structure onto unmodified human WT α-syn. α-Syn was iteratively seeded and the amyloid structure analyzed by PK digestion. **h**, α-Syn(gS87) PFFs can seed the aggregation of unmodified mouse α-syn. α-Syn PFFs (unmodified or gS87) were added to buffer or unmodified mouse α-syn (50 μM monomer concentration, 5% PFF) before aggregation and analysis by sedimentation and Coomassie staining. The results are mean ± s.e.m. of experimental replicates (*n* = 3). Statistical significance was determined using a one-way ANOVA test followed by Tukey’s multiple comparison test. **i**, α-Syn(gS87) can be phosphorylated at serine 129 (pS129). The indicated α-syn PFFs or monomers were incubated with PLK3, and pS129 on α-syn was visualized by WB using pS129 antibody (81a). **j**, The O-GlcNAc on α-syn(gS87) is resistant to removal by OGA post-aggregation. α-Syn(gS87) monomers or PFFs were treated with OGA for the indicated amounts of time, and the removal of O-GlcNAc was measured using RP-HPLC. The results are mean ± s.e.m. of experimental replicates (*n* = 3). Source data

As previously described^35^, we prepared α-syn(gS87) through an expressed portion ligation (EPL)^36,37^ strategy of iterative ligation reactions and a final desulfurization step (Fig. 1c,d). We previously demonstrated that monomeric α-syn(gS87) forms fibrillar aggregates at a low concentration (50 μM), with reduced kinetics compared with the unmodified protein^35^. Others have shown that higher concentrations of α-syn facilitate the production of fibrils, hereafter referred to as preformed fibrils (PFFs), in large amounts for subsequent in vitro, neuron culture and in vivo seeding experiments. Therefore, we separately subjected α-syn or α-syn(gS87) to the well-established and validated α-syn fibrillization conditions (α-syn for 7 days in phosphate-buffered saline (PBS))^38^. As expected, α-syn(gS87) resulted in indistinguishable levels of thioflavin T (ThT) signal at both concentrations (Fig. 2a). ThT binding alone is sometimes not sufficient to allow for direct comparison of fibril formation because amyloid fibrils can bind ThT differently, yielding higher or lower signals^39,40^. We then also analyzed the aggregation reactions by sedimentation and sodium dodecyl sulfate–polyacrylamide gel electrophoresis (SDS–PAGE) (Fig. 2b). These data confirmed that α-syn(gS87) aggregates but to a lesser extent, consistent with our previous finding that O-GlcNAc inhibits the nucleation step of α-syn aggregation^35^.

We then confirmed that both α-syn and α-syn(gS87) are forming amyloid fibrils using transmission electron microscopy (TEM) (Fig. 2d). To indirectly probe for structural differences, we subjected unmodified and α-syn(gS87) to proteinase K (PK) digestion. Briefly, PK readily digests monomeric α-syn to very small peptide fragments; however, it cannot gain access to the core of amyloid fibrils, resulting in limited proteolysis. We visualized the PK digestion reactions and found the expected five bands associated with the typical PFF structure of α-syn, while observing only three bands from α-syn(gS87) PFFs (Fig. 2e). Together, these results show that α-syn(gS87) can indeed form an amyloid strain that differs from those formed by the unmodified protein.

### α-Syn(gS87) seeds aggregation for cellular and in vivo analyses

Next, we asked whether α-syn(gS87) PFFs could seed the aggregation of unmodified protein. We first generated either α-syn or α-syn(gS87) PFFs by incubation of the monomeric forms of these proteins for 7 days (172 μM). Next, we compared the ability of the two PFF preparations to seed the aggregation of human unmodified α-syn (50 μM with 5% PFFs) in vitro. The efficiency of seeding was assessed by monitoring the kinetics of aggregation by sedimentation as above and found that α-syn(gS87) PFFs do seed monomeric protein, and the extent of fibrillization is indistinguishable by 24 h (Fig. 2f and Supplementary Fig. 1). We subjected a portion of the α-syn(gS87) seeded aggregation reaction to PK digestion and found that α-syn(gS87) PFFs template their amyloid structure onto unmodified protein (Fig. 2g), and this templating behavior is maintained over multiple rounds of seeding (Fig. 2g).

**Extended Data Fig. 1:**
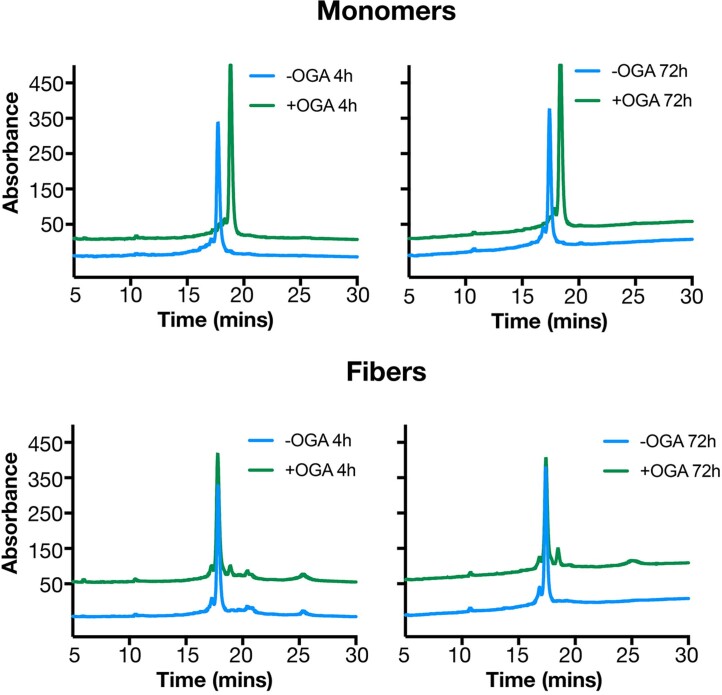
The O-GlcNAc on α-Syn(gS87) fibers is stable to enzymatic removal. α-Syn(gS87) monomers or PFFs (25 uM) were incubated with bacterial O-GlcNAc hydrolase BtGH84 (1 uM) for the indicated lengths of time at 37 °C in phosphate buffered saline. The reactions were solubilized in 8 M urea and analyzed by RP-HPLC. The identity of the O-GlcNAc modified and deglycosylated proteins were confirmed by ESI-MS.

PFF treatment of mouse neurons or injection of α-syn PFFs into mice brains represent standard approaches to evaluate PFF pathogenicity, toxicity and propagation^38^. However, previous studies have shown that human α-syn PFFs seed mouse α-syn monomers less efficiently^7,12,41^. This raised the possibility that our human α-syn(gS87) PFFs may not seed the aggregation of mouse α-syn. Therefore, we tested whether α-syn(gS87) PFFs can seed mouse α-syn aggregation in vitro and found that they can and reach similar levels after only 12 h (Fig. 2h and Supplementary Fig. 2). We and others have also shown that the seeding of endogenous α-syn aggregation in neurons results in phosphorylation of the newly seeded aggregates at serine 129 (pS129) (ref. ^7^), as observed in the human PD pathology. This enables pS129 to be used as a surrogate marker for pathology formation and propagation in neurons and in vivo. We wondered whether the α-syn(gS87) aggregate structure might be refractory to phosphorylation. Since this structure is templated (Fig. 2g), it might prevent us from visualizing neuronal seeding by α-syn(gS87) PFFs using a pS129 antibody. However, we found that α-syn(gS87) PFFs can be phosphorylated by PLK3, a kinase that contributes to ps129, indicating that we can still use pS129 as a pathology mark (Fig. 2i). Finally, because O-GlcNAc is a dynamic modification, we were concerned that cellular OGA might remove O-GlcNAc once the PFFs were taken up into neurons. As expected, when we treated α-syn(gS87) monomers with OGA in vitro, we observed rapid loss of the O-GlcNAc (Fig. 2j and Extended Data Fig. 1). However, O-GlcNAc on the α-syn(gS87) PFFs was quite stable, even after 72 h of OGA treatment (Fig. 2j and Extended Data Fig. 1), suggesting that the region around S87 cannot bind to OGA in the required extended confirmation^42,43^. These data show that α-syn(gS87) PFFs seed and template their structure onto monomeric, unmodified protein and can be analyzed using mouse neurons and brains.

### α-Syn(gS87) PFFs induce significantly less seeding and pathology

**Fig. 3:**
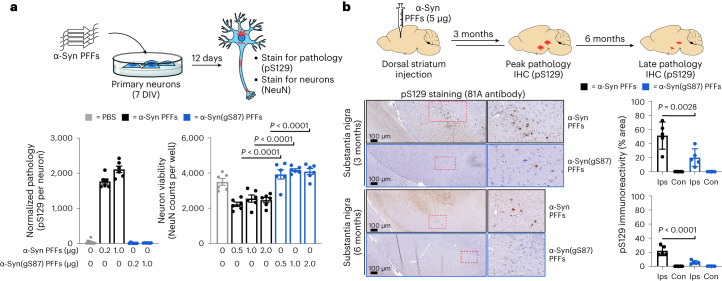
α-Syn(gS87) PFFs have diminished ability to induce pathology formation, spreading and toxicity in cells and in vivo. **a**, Primary embryonic hippocampal neurons at 7 DIV were treated with the indicated concentrations of PFFs or PBS for 12 days before analysis of pathology (pS129 staining) and neuron viability (NeuN staining). The results are mean ± s.e.m. of biological replicates (*n* = 6). Statistical significance was determined using a one-way ANOVA test followed by Sidak’s multiple comparison test. **b**, WT mice were injected with α-syn or α-syn(gS87) PFFs (5 μg) in a single unilateral injection into the dorsal striatum. Pathology was visualized by immunohistochemistry (IHC) against pS129 at 3 and 6 months post-injection. Results are mean ± s.e.m. of biological replicates (*n* = 5). Statistical significance was determined using a one-way ANOVA test followed by Tukey’s multiple comparison test. Ips, ipsilateral and con, contralateral. Source data

Next, we investigated the effect of O-GlcNAc modification at S87 on α-syn PFFs-mediated induction of α-syn fibrillization and formation of Lewy body (LB)-like inclusions using the well-established seeding-dependent neuronal model of α-syn pathology formation^5^. Specifically, hippocampal primary neurons from mouse embryos were treated with different concentrations of either α-syn or α-syn(gS87) PFFs for 12 days. We then measured the extent of PFFs-mediated seeding of α-syn aggregation and neuron viability using fluorophore-labeled antibodies against pS129 and NeuN, respectively (Fig. 3a). Consistent with previous data, α-syn PFFs induced robust seeding activity and aggregation of endogenous α-syn, and neuron death. In stark contrast, we did not observe any increase in pS129 signal or neuron death upon treatment with syn(gS87) PFFs.

**Extended Data Fig. 2:**
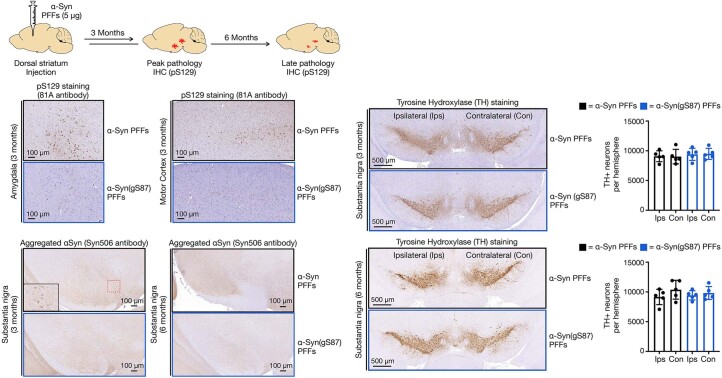
α-Syn(gS87) PFFs have diminished pathology without loss of dopaminergic neurons. Wild-type mice were injected with α-Syn or α-Syn(gS87) PFFs (5 μg) in a single unilateral injection into the dorsal striatum. Pathology was visualized by immunohistochemistry against pS129 or aggregated αSyn at 3 or 6 months post-injection. Loss of dopaminergic neurons was visualized by immunohistochemistry against tryrosine hyroxlyase (TH). Results are mean ± SEM of biological replicates (n = 5). Statistical significance was determined using a Paired Student’s T-test, and no statistical differences between ipsilateral and contralateral sides were found. Source data

Given the striking nature of this difference, we sought to validate these findings in vivo. We performed a dorsal striatum injection of either α-syn or α-syn(gS87) PFFs (5 μg) into the brains of wild-type (WT) C57Bl6/C3H mice. After 3 and 6 months, we euthanized the mice and stained for pS129 as a marker for α-syn aggregation and Lewy body pathology. In this model, injection of human α-syn PFFs results in peak pathology at around 3 months that diminishes over time and no significant loss of tyrosine–hydroxylase-positive neurons in the substantia nigra^12^. Injection of α-syn(gS87) PFFs resulted in dramatically less overall pS129 staining in the substantia nigra and significantly less area with pS129-positive inclusions when compared with unmodified PFFs (Fig. 3b). We found the same difference in other parts of the brain including the amygdala and motor cortex (Extended Data Fig. 2). We also confirmed the difference in pathology in the substantia nigra using an antibody (α-syn506) against aggregated α-syn (Extended Data Fig. 2). Additionally, we observed no significant loss of tyrosine–hydroxylase-positive neurons in mice injected with either α-syn or α-syn(gS87) PFFs (Extended Data Fig. 2), as expected with human PFFs. These data demonstrate that α-syn(gS87) PFFs are less prone to induce pathology in neurons and in vivo. They also set up an interesting divergence between our in vitro data where α-syn(gS87) PFFs can seed additional aggregation (Fig. 2f–h) and in neurons where they exhibit dramatically reduced seeding activity.

### Unmodified and α-syn(gS87) PFFs are handled similarly by neurons

The spread of α-syn PFFs and induction of further aggregation and toxicity is a multistep process^7,44^. In culture, PFFs are first taken up by neurons through the endosomal/lysosomal pathway. After they gain access to the cytosol, the PFFs are cleaved by calpain (residues 114 and 122 in vitro)^6,7,45^ and potentially other proteases to generate truncated amyloids with a monomeric molecular weight of ~12 kDa, down from the 15 kDa of full-length α-syn. These truncated PFFs seed the aggregation of endogenous α-syn, which is then phosphorylated to give the pS129 mark associated with pathology. Finally, the aggregates mature into LB-like structures made up of proteins, lipids, membranous structures and organelles. This overall pathway is associated with neuron dysfunction and cell death^7,46^. We reasoned that a better understanding of α-syn(gS87) PFFs structural properties, internalization, processing and seeding in neurons could provide hints as to why they can seed and template aggregation in vitro but apparently not in neurons or brains.

**Fig. 4:**
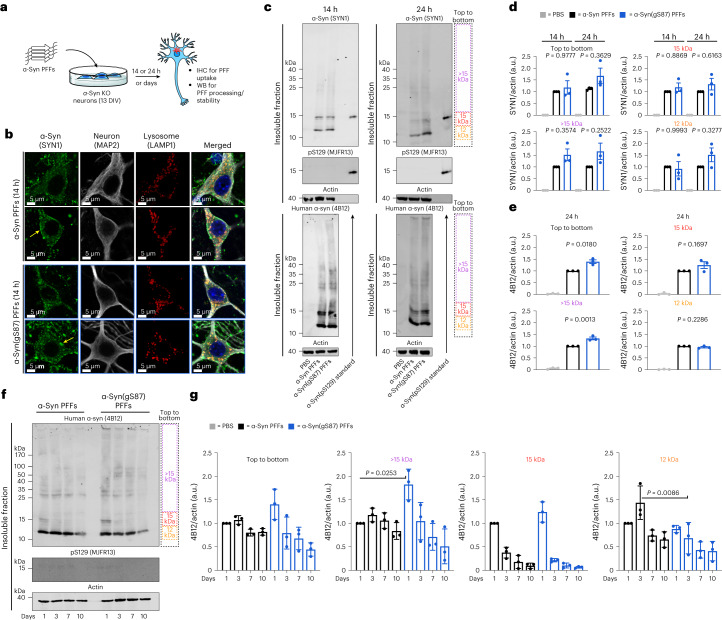
Unmodified and α-syn(gS87) PFFs display similar uptake, processing and stability in neurons. **a**, Experimental outline. Primary hippocampal neurons from α-syn KO mice at 13 DIV were treated with the indicated PFFs (70 nM) or PBS for different lengths of time before the following analyses. **b**, O-GlcNAc at S87 does not alter the internalization of PFFs as visualized by ICC after 14 h of treatment. The results are consistent between biological replicates (*n* = 3). **c**, O-GlcNAc at S87 does not notably affect the internalization, C-terminal cleavage to ~12 kDa fragment or phosphorylation at S129 (pS129) of PFFs as visualized by WB after 14 or 24 h of treatment. **d**, Quantitation of the data in **c** using the pan-α-syn antibody SYN1. The results are mean ± s.e.m. of biological replicates (*n* = 3). **e**, Quantitation of the data in **c** using the human-specific α-syn antibody 4B12. The results are mean ± s.e.m. of biological replicates (*n* = 3). **f**, O-GlcNAc at S87 does not notably alter the stability of internalized PFFs as visualized by WB over 10 days of treatment. **g**, Quantitation of the data in **f**. The results are mean ± s.e.m. of biological replicates (*n* = 3). In all experiments, statistical significance was determined using a one-way ANOVA test followed by Tukey’s multiple comparison test. Source data

**Extended Data Fig. 3:**
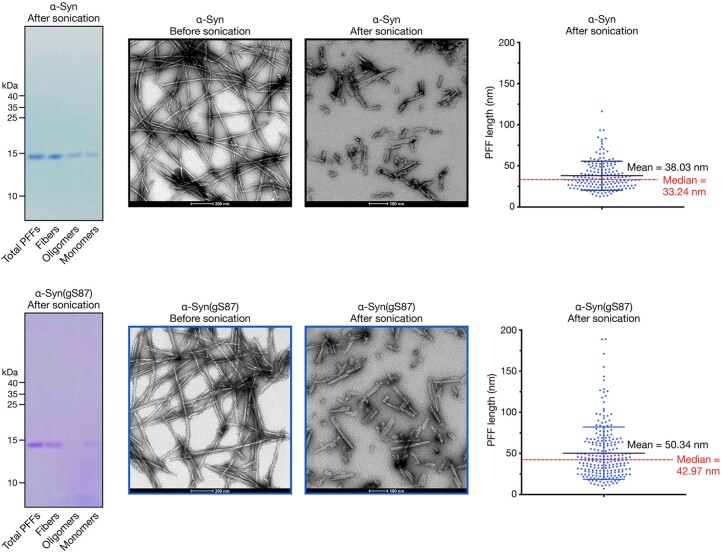
Characterization of PFFs. α-Syn or α-Syn(gS87) were subjected to aggregation conditions (172 μM, 7d). Fibers, oligomers, and monomers contained in these aggregation reactions were then separated [Kumar et al. *J Neurochem* 153, 103–119 (2020)]. The overall aggregation reaction (Total PFFs) and the individual components were analyzed by Coomassie staining. The corresponding PFFs were also visualized before and after sonication and measured after sonication using transmission electron microscopy (TEM).

First, in-depth characterization of the unmodified α-syn and α-syn(gS87) PFFs revealed that they are quite similar in that they are both made up of primarily fibrils over oligomers and are of a similar length distribution suitable for uptake by neurons (Extended Data Fig. 3). To determine whether the two types of fibril are differentially taken up by neurons, we quantified their uptake in primary neurons from α-syn knockout (KO) mice^6,7^, as they allow us to specifically investigate the fate of exogenous unmodified and α-syn(gS87) PFFs, without confounding issues due to the presence or aggregation of the endogenous protein (Fig. 4a).

We assessed the uptake of PFFs by treating these neurons with α-syn or α-syn(gS87) PFFs (70 nM) for 14 h and examined their localization using immunocytochemistry (ICC) (Fig. 4b). As expected^6,47^, we found that unmodified PFFs and α-syn(gS87) PFFs accumulated on cellular membranes (yellow arrows) and were taken up into neurons and appeared as puncta structure (Fig. 4b). Both PFFs seeds strongly colocalized with the lysosomes (LAMP1-positive vesicles) (Fig. 4b), indicating that the O-GlcNAc modification does not influence the internalization of the α-syn PFFs via the endosomal/lysosomal pathway. Next, we investigated whether α-syn(gS87) PFFs were processed differently by treating α-syn KO neurons for 14 or 24 h. Western blot (WB) analysis of the insoluble fraction of the PFF-treated neurons was performed using both pan (SYN1) and human-specific (4B12) α-syn antibodies. Figure 4c confirmed the internalization of the PFFs in the neurons as indicated by the presence of SDS-resistant aggregates above 15 kDa (higher molecular weights (HMWs), >15 kDa) to a similar level (Fig. 4d,e) for each type of PFFs. As previously described^6,7^, we observed the accumulation of a 12 kDa fragment, and these seeds were not phosphorylated at S129 (pS129) following their C-terminal cleavage (Fig. 4c). Notably, we observed no differences in the proteolytic processing or phosphorylation of the α-syn(gS87) PFFs (Fig. 4c). To confirm these observations, we performed multiple biological replicates (Supplementary Fig. 3) and quantified the signal from different regions (Fig. 4d). We found no significant differences between α-syn(gS87) PFFs using the pan-antibody (SYN1) and only small differences with the human-specific antibody (4B12). Mouse PFFs were used as a positive control in all of our experiments, and no notable differences in terms of internalization and processing were observed compared with the unmodified and α-syn(gS87) PFFs (Supplementary Fig. 3). Finally, we sought to explore whether the two types of PFFs were differentially cleared once internalized into neurons. Unmodified and α-syn(gS87) PFFs (70 nM) were added to α-syn KO neurons, and their fate was monitored for up to 10 days by measuring the amount of remaining PFFs by WB (Fig. 4f). We observed a trend where the internalized α-syn(gS87) PFFs appear to be cleared faster than the unmodified α-syn(gS87) PFFs (Fig. 4g and Supplementary Fig. 4). Together, these results demonstrate that O-GlcNAc modification of α-syn does not alter uptake or proteolytic processing of the PFFs, but may promote their clearance in neurons.

### α-Syn(gS87) PFFs seed far less aggregation in neurons

**Fig. 5:**
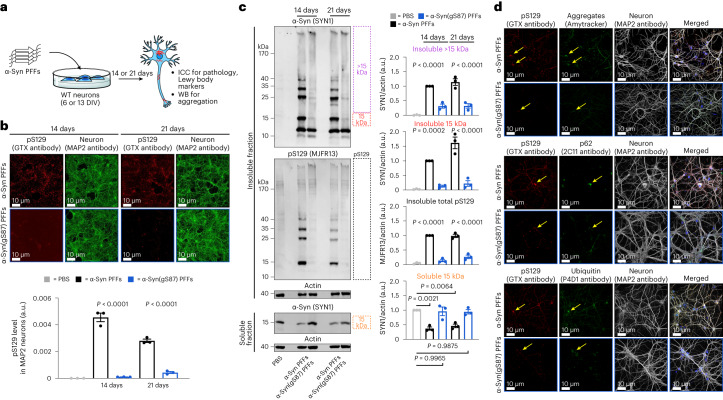
α-Syn(gS87) PFFs have dramatically reduced seeding capacity in neurons. **a**, Experimental outline. Primary hippocampal neurons from WT mice at 6 or 13 DIV were treated with the indicated PFFs (70 nM) or PBS for 21 and 14 days, respectively, before the following analyses. **b**, O-GlcNAc at S87 notably reduced the formation of pS129-positive aggregates in neurons as visualized and quantified using ICC combined with high content imaging. **c**, Unmodified PFFs seed the aggregation of endogenous α-syn into insoluble and pS129-positive higher molecular-weight aggregates. O-GlcNAc at S87 dramatically reduced this seeded aggregation, and more endogenous α-syn remained soluble. The results shown in **b** and **c** are the mean ± s.d. of biological replicates (*n* = 3). Statistical significance was determined using a one-way ANOVA test followed by Tukey’s multiple comparison test. **d**, Aggregates that form from α-syn(gS87) PFFs are notably reduced but display Lewy body hallmarks (amyloid, p62 and ubiquitination) by ICC. The results are consistent between biological replicates (*n* = 3). Source data

We next examined whether O-GlcNAc modification of α-syn PFFs influences their ability to seed the aggregation of endogenous α-syn in neurons. Specifically, we treated primary neurons from WT mouse pups with α-syn or α-syn(gS87) PFFs (70 nM) for 14 or 21 days and examined the extent of seeded aggregation using ICC and WB analyses (Fig. 5a)^7^. Consistent with our data in Fig. 3a, we again observed almost no detectable α-syn(gS87) PFF-induced pS129 staining after 14 days and significantly less at 21 days compared with unmodified PFFs. These two sets of results (Figs. 3a and 5b), obtained by independent researchers at different locations and using two different model systems (embryonic versus postnatal neurons), confirm that α-syn(gS87) PFFs exhibit diminished seeding activity and pathology as measured by pS129. We then isolated the insoluble and soluble fractions of the neurons and analyzed them by WB (Fig. 5c and Supplementary Fig. 5). We observed a ladder of protein species detected by both anti-α-syn and anti-pS129 antibodies in neurons treated with unmodified PFFs, consistent with the partial stability of the seeded aggregates to SDS. In contrast, we found very little laddering and a much lower overall WB signal from neurons treated with α-syn(gS87) PFFs. Quantitation of the blots confirmed that α-syn(gS87) PFFs seed significantly less aggregation of endogenous α-syn (Fig. 5c). We also confirmed that mouse PFFs induce an indistinguishable WB pattern compared with human unmodified PFFs (Supplementary Fig. 5), as previously described^46^. We also analyzed the portion of α-syn that remained in the soluble-protein fraction (Fig. 5c and Supplementary Fig. 5). We found that unmodified α-syn PFFs result in the loss of soluble α-syn as it is consumed by the seeded aggregation process. However, in the case of α-syn(gS87) PFF treatment, we observed essentially no significant loss of soluble α-syn, consistent with their diminished seeding activity in neurons.

**Extended Data Fig. 4:**
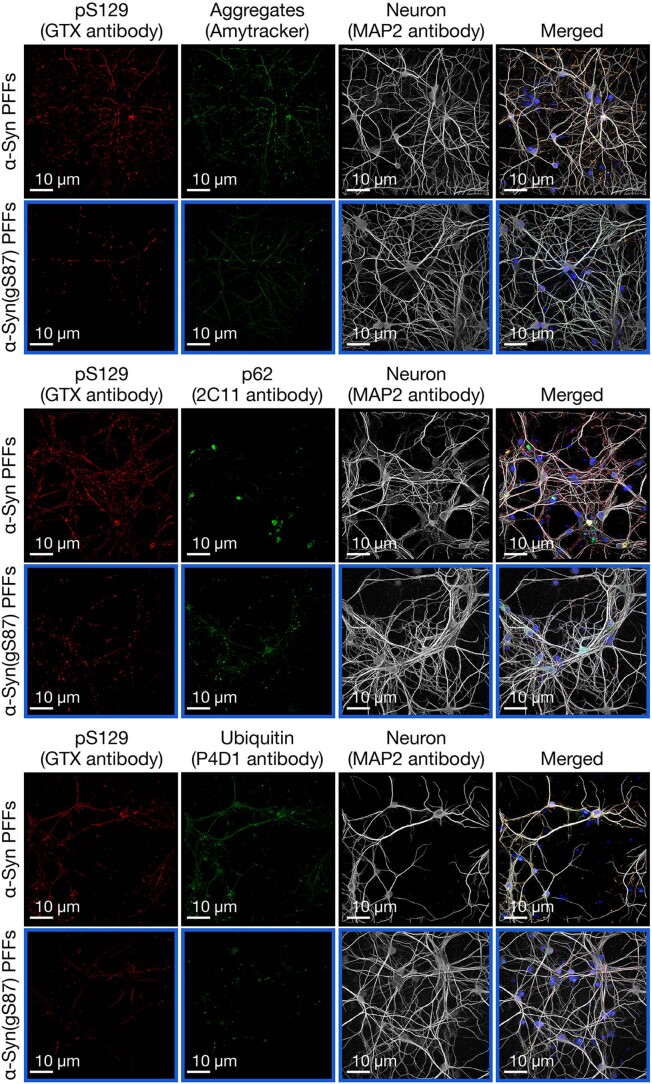
Aggregates that form from α-Syn(gS87) PFFs are notably reduced but display Lewy body hallmarks. Primary hippocampal neurons from wild-type mice at 7 days in vitro (7 DIV) were treated with the indicated PFFs (70 nM) or PBS for 14 d before visualization of aggregate markers (amyloid, p62 & ubiquitination) by immunocytochemistry (ICC). Results are consistent between biological replicates (n = 3).

We then investigated, by ICC and confocal imaging, whether the seeded aggregates formed in α-syn(gS87) PFFs-treated neurons were differently stained by the well-established LB markers, including the autophagy marker p62, ubiquitin and the Amytracker amyloid-like specific dye (Fig. 5d and Extended Data Fig. 4). The few pS129-positive seeded aggregates formed in the α-syn(gS87) PFFs-treated neurons all positively stained for p62, ubiquitin and the Amytracker dye, indicating that when these aggregates do form, they share the same hallmarks of the seeded aggregates formed in the unmodified PFFs-treated neurons. This observation suggests that α-syn(gS87) PFFs do not alter the morphological diversity of LB-like inclusions, as was recently observed for the human α-syn E83Q PFFs^46^. We also observed that when the aggregates were formed in the α-syn(gS87) PFFs-treated neurons, they mostly localized in the soma. Barely any aggregates were observed in the neurites of these neurons compared with the extensive neuritic pathology observed from unmodified PFFs (Fig. 5d).

Overall, these data show that, although α-syn(gS87) PFFs can seed aggregation of unmodified α-syn in vitro, they exhibit diminished seeding activity in living neurons. Additionally, they indicate that this is the main distinguishing feature between the two classes of PFFs, although the difference in stability seen in the KO neurons may also contribute.

### Altered fibril interactions could contribute to diminished seeding

**Extended Data Fig. 5:**
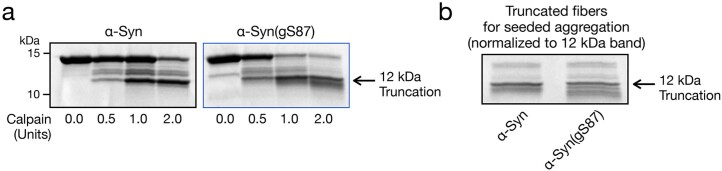
Generation of truncated α-Syn PFFs. **a**) α-Syn or α-Syn(gS87) were subjected to aggregation conditions (172 μM, 7 d) before incubation with the indicated amounts of calpain and analysis by SDS-PAGE and Coomassie staining. Cleavage experiments were repeated on 2 batches of aggregates with similar results. **b**) The levels of PFFs truncated by 2.0 units of calpain were normalized by Coomassie staining for use in seeded aggregation experiments. Shown is a representative analysis of a normalization experiment, and normalization was performed for every batch of truncated PFFs used in seeding experiments.

**Extended Data Fig. 6:**
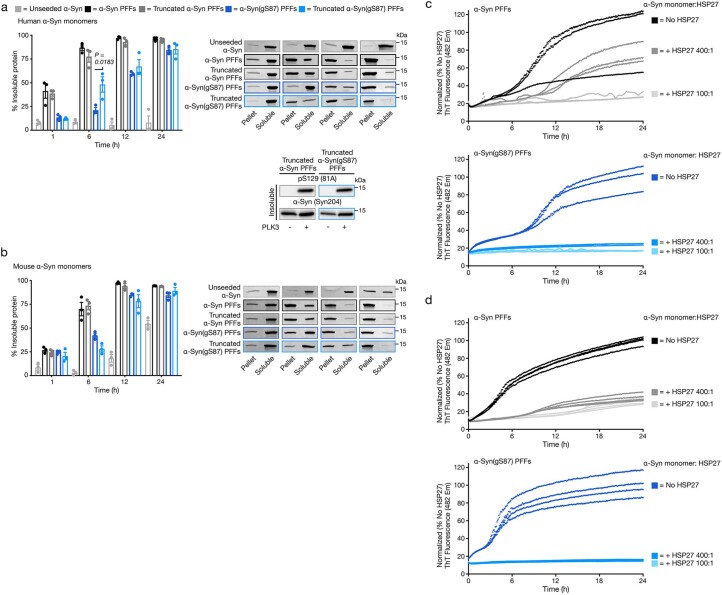
Altered protein interactions rather than inherent seeding-capacity likely explain the divergence between α-Syn(gS87) PFFs *in vitro* and *in vivo*. **a**) Truncation does not dramatically alter the seeding of human α-synuclein aggregation by unmodified or α-Syn(gS87) PFFs can seed aggregation of unmodified, human α-synuclein. α-Syn PFFs (unmodified or gS87) were generated by aggregation of unmodified protein (172 μM) followed by sonication. The corresponding truncated PFFs were generated by digestion enzymatic digestion of PFFs with calpain (Extended Data Fig. 5). The resulting PFFs were then added to buffer or unmodified, human α-Syn (50 μM monomer concentration, 5% PFF) before aggregation and analysis by sedimentation and Coomassie staining. Results are mean ± SEM of experimental replicates (n = 3). Statistical significance was determined using a one-way ANOVA test followed by Tukey’s multiple comparison test. The insoluble fractions at 24 h were then subjected to phosphorylation by PLK3 and pS129 was visualized by WB. **b**) PFFs were generated as in (a) were then added to buffer or unmodified, mouse α-synuclein (50 μM monomer concentration, 5% PFF) before aggregation and analysis by sedimentation and Coomassie staining. Results are mean ± SEM of experimental replicates (n = 3). **c**) HSP27 more potently inhibits aggregation seeded by α-Syn(gS87) PFFs. α-Syn monomers (50 μM) and the indicated ratios of HSP27 were mixed with the indicated PFFs (2.5 μM, 5%). The reactions were placed in a plate reader and aggregation was detected by ThT fluorescence (λ_ex_ = 450 nm, λ_em_ = 482 nm). **d**) Replicate experiment of (c). Source data

We next set out to test potential molecular mechanisms to reconcile the in vitro and in vivo seeding capacity of α-syn(gS87) PFFs. Given that the C-terminal domain of α-syn decorates the surface of PFFs, we wondered whether cleavage of the C-terminus of internalized α-syn(gS87) PFFs might interfere with their ability to seed the aggregation of endogenous α-syn. To directly test this possibility in vitro, we generated PFFs and subjected them to different amounts of calpain and analyzed the production of the 12 kDa fragment by SDS–PAGE (Extended Data Fig. 5a). We then normalized the amounts of 12 kDa α-syn and α-syn(gS87) PFFs (Extended Data Fig. 5b) and used them to seed unmodified human α-syn aggregation. We observed very little difference in seeded aggregation between the full-length WT and α-syn(gS87) PFFs and the corresponding 12 kDa PFFs (Extended Data Fig. 6a), and the aggregates formed from both truncated PFFs could still be phosphorylated at S129 by PLK3 (Extended Data Fig. 6a). We also made the same observation concerning seeded aggregation with mouse monomeric α-syn (Extended Data Fig. 6b). These results suggest that the cleavage of PFFs in neurons cannot explain the lack of seeded aggregation for α-syn(gS87); however, they show directly for the first time, to our knowledge, that postfibrilization cleavage of PFFs (even unmodified ones) does not greatly influence their seeding activity in vitro.

We next reasoned that the lack of seeding in neurons and in vivo may result from altered protein interactions between the two types of PFFs, with other proteins including chaperones. The small heat shock proteins (sHSPs)^48^ represent one such interaction that can directly inhibit PFF seeding^49^. We hypothesized that sHSPs may more potently inhibit α-syn(gS87) PFF seeding compared with unmodified PFFs and directly tested this possibility with heat shock protein 27 (HSP27), an sHSP highly expressed in the brain. Specifically, we performed seeded aggregation experiments, with either α-syn or α-syn(gS87) PFFs and unmodified monomer, in the presence of different ratios of HSP27 (Extended Data Fig. 6c,d). Consistent with our previous results^50^, HSP27 displayed partial to full inhibition of aggregation seeded by α-syn PFFs at different ratios. Strikingly, HSP27 displayed more potent inhibition of seeding by α-syn(gS87) PFFs. These results confirm our hypothesis, at least in vitro, and strongly suggest that α-syn(gS87) PFFs are more effectively chaperoned in neurons and most probably have other different protein interactions that prevent seeded aggregation and the development of pathology in vivo.

### Structure of α-syn(gS87) amyloids are different from other fibrils

**Fig. 6:**
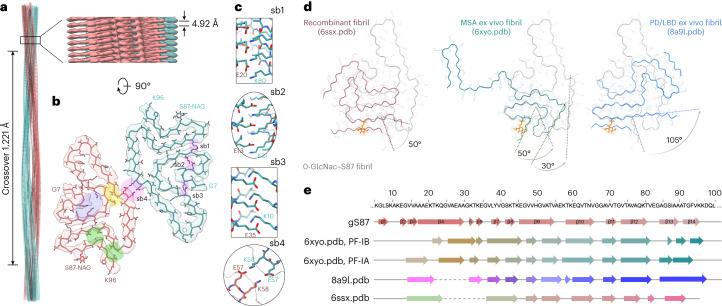
Cryo-EM structure and atomic model of the gS87 double filaments and comparison to other fibril structures. **a**, The cryo-EM structure (PBD 8GF7) of full-length gS87 fibril with a crossover distance of 1,221 Å, and a close-up side view of the reconstructed 3D map showing the distance between layers, helical rise, of 4.92 Å. **b**, A cross-section of the density map (salmon and green) overlaid with the atomic model. Areas highlighted in pink indicate potential salt bridges. Areas highlighted in yellow and green indicate hydrophobic interdigitated interfaces. The area highlighted in purple indicates a lysine-rich region. **c**, Five possible salt bridges connecting the two protofilaments (sb1), and within one protofilament (sb2, sb3 and sb4). **d**, Superimposition of the gS87 protofilament (light gray ribbon) to an unmodified in vitro fibril (red ribbon, pdf: 6ssx), a MSA ex vivo fibril (green ribbon, PDB: 6xyo) and a PD/LBD ex vivo fibril (blue ribbon, PDB: 8a9l). **e**, Secondary structural comparison of gS87 double filaments to MSA ex vivo fibril (protofilament IA, protofilament IB, PDB: 6xyo) and PB/LBD ex vivo fibril (PDB: 8a9l) with primary sequence indicated on the top.

**Extended Data Fig. 7:**
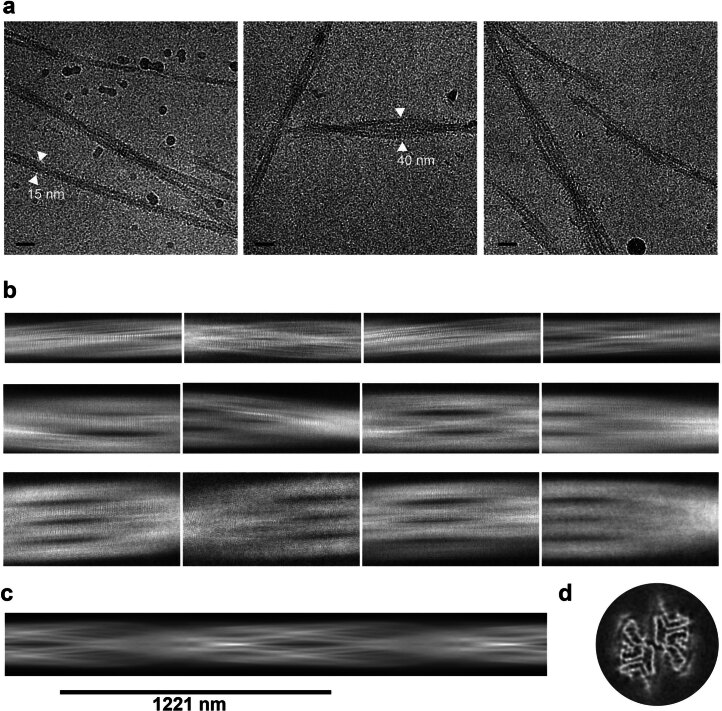
Cryo-EM data collection and processing of α-Syn(gS87) fibrils. **a**) Representative cryo-EM micrographs showing polymorphisms of α-Syn(gS87) fibrils and their estimated width. Scale, 200 nm. These data representative of 4 experimental replicates. **b**) Representative 2D class averages of different morphologies, double filaments – top row, triple filaments – middle row and quadruple filaments. **c**) Initial model of α-Syn(gS87) fibril generated by Relion3.1. **d**) 3D class average of α-Syn(gS87) fibril.

Finally, we set out to determine whether O-GlcNAc alters the structure of α-syn. Toward this goal, we sought to determine the structure of α-syn(gS87) fibrils using cryo-EM. We subjected α-syn(gS87) to aggregation and used TEM and cryo-EM to visualize fibrils, revealing various fibril morphologies, including double, triple and quadruple filaments comprised of two, three and four protofilaments, respectively (Extended Data Fig. 7a,b). The most abundant species was the double filament, which had a diameter of approximately 20 nm. The triple and quadruple filaments had diameters of approximately 30 nm and 40 nm, respectively. We collected cryo-EM images and manually picked all filaments for data processing and structure determination. Due to the limited number of segments present in the triple and quadruple filament morphologies, we only performed three-dimensional (3D) reconstruction with the double filaments. The 3D classification resulted in one main class with two protofilaments seemingly related by a twofold axis. The resulting density map, with a resolution of 4.97 Å, corresponded to a crossover of 1,221 Å, an optimized twist angle of −0.7° (assumed to be left handed), and a helical rise of 4.92 Å (Fig. 6a).

**Extended Data Fig. 8:**
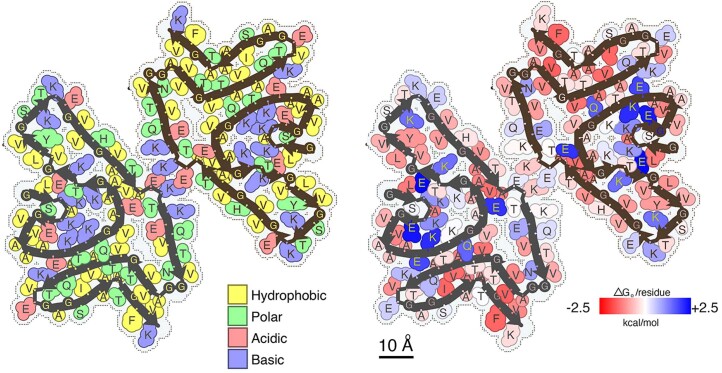
Residues composition (left) and solvation energy map (right) of the α-Syn(gS87) double filaments. Residues on the left are colored by hydrophobicity (yellow: hydrophobic, green: polar, red: acidic, and blue: basic). Residues on the right are colored from favorable (red, −2.5 kcal/mol) to unfavorable stabilization energy (blue, 2.5 kcal.mol).

Modeling structures at this limited resolution cannot necessarily define molecular features, but the density map could fit a reasonable α-syn(gS87) model comprising the residues from glycine 7 to lysine 96 and an extra density corresponding to the sugar on serine 87. This analysis shows several salt bridges potentially holding together the two protofilaments (Fig. 6b,c, sb1) and contributing to the folding of the α-syn(gS87) fiber structure (Fig. 6b,c, sb2, sb3 and sb4). Additionally, this model suggests that the hydrophobic interfaces are relatively small compared with other amyloid fibrils (Fig. 6b, highlighted in yellow and green), which may explain some of the difference in fibril stability we observed, as they would have a solvation energy of approximately −24.0 kcal mol^−1^ (Extended Data Fig. 8)^15,51^

Comparison of the model to disease fibrils shows that α-syn(gS87) fibrils share some structural similarities with fibrils obtained from individuals with MSA and PD/LBD^17,52^, but that the O-GlcNAc is incompatible with the conformation found in the MSA or PD/LBD ex vivo fibrils (Fig. 6d). We also compared α-syn(gS87) fibrils to the amyloid strain formed in vitro under our aggregation conditions^53^ and again found that the O-GlcNAc is incompatible with the structure (Fig. 6d, left). The secondary structure composition of the compared models is detailed in Fig. 6e. The electron density confirms that α-syn(gS87) fibrils are quite different from both the pathogenic unmodified fibrils we prepared here and the amyloids isolated from patient brains to date, and our structural model potentially explains how O-GlcNAc statically forces a different fold. Our results suggest that PTMs contribute to expanding the conformational landscape of amyloid fibrils and are key determinants of amyloid structural diversity.

Our results demonstrate that O-GlcNAc can cause the formation of an alternative strain of α-syn(gS87) amyloids with diminished in vivo seeding activity. Interestingly, although α-syn(gS87) PFFs can readily seed additional aggregation in vitro and template their structure onto unmodified α-syn, they display very low levels of seeding activity in neurons or in vivo. In the full-length fibrils, O-GlcNAc is stable against removal by OGA. This is consistent with its position in the core of the fibril structure, which would be unlikely to be dynamic enough to bind in the required extended conformation to the OGA active site. We do not necessarily know if this is the case after C-terminal cleavage, but the difference in seeded aggregation remains the same in either case. These observations suggest that the effect of this modification, not only on the structure of the fibrils but also on how they interact with other molecules, proteins and organelles in the cellular environment, is a key determinant of their seeding activity in vivo. We reasoned that O-GlcNAc may alter the α-syn–fibril interactome in ways that favor interaction with chaperones or other proteins that could modify the surfaces of the fibrils and inhibit their seeding activity and confirmed this hypothesis in vitro with HSP27. We think that it is very likely that other proteins may also be responsible and plan to explore how the overall interactome might be altered in the future. Finally, we took advantage of cryo-EM to generate a model of the α-syn(gS87) PFFs that appears to be an amyloid strain that is very different from the unmodified PFFs used here as well as aggregates analyzed from synucleinopathy patient samples. We speculate that any of these features may allow HSP27, and probably other protein factors, to recognize the amyloid core and/or the display of the N- and C-terminal extensions or ‘fuzzy coat’ of the protein in ways that could also be differentiated from the unmodified PFFs. Our findings also show that not all the fibrils in the brain are pathogenic and suggest that correlating fibril/pathology load in the brain and neurodegeneration of clinical symptoms may not be the best approach to assessing the efficacy of therapeutics in clinical trials. This underscores the critical importance of developing diagnostics and therapeutics that account for not only the structural diversity of fibrils but also their differential pathogenic properties.

Taken together, we believe that these results have important implications for amyloidogenesis in general and the exploitation of O-GlcNAc in the treatment of PD and other NDDs. To our knowledge, this is the first example of a PTM that occurs on α-syn monomers that can cause the formation of an amyloid strain that shows such dramatically reduced pathogenicity. Additionally, the divergence between our in vitro and cellular seeding results is striking. These data suggest that interactions between α-syn fibrils and other cellular proteins or organelles are key determinants of their pathology formation and toxicity. They show that connections between the in vitro seeding and pathogenic potential of different amyloids might be decoupled and should be considered in future experiments. Our results also support the continued development of OGA inhibitors to treat PD, as increased O-GlcNAc has the strong potential to both slow the initial aggregation of α-syn monomers but also result in the formation of in vivo seeding-incompetent fibrils. This is particularly true given the inconsistent results of recent antibody therapies that target the amyloid aggregates. Two compelling hypotheses for the failure of some of these drugs are that loss of the monomeric protein and/or the aggregation process overall are the underlying toxic events. The results here, combined with our previous publications, provide a strong foundation that increased O-GlcNAc will maintain the levels of soluble α-syn and slow the aggregation process through the direct inhibition of the initiation of aggregation^34,35^ and reduced seeding. This might also be true for O-GlcNAc on other proteins such as tau, as the major site of O-GlcNAc (S400) also slows but does not completely block its aggregation^33^.

## Methods

### General

Solvents and reagents obtained from commercial sources were used without any further purification. Aqueous solutions were prepared using ultrapure water from an in-house water purification (reverse osmosis and deionization) system. Bacterial growth media and standard buffers were prepared and sterilized according to the instructions of the manufacturer without custom modifications. Bacterial growth media and cultures were handled under sterile conditions. Quantifications of protein concentrations were performed using the Pierce bicinchoninic acid assay (BCA) kit (Thermo Fisher Scientific). Reversed-phase high-performance liquid chromatography (RP-HPLC) was performed with Agilent 1200 Series HPLC instruments equipped with a diode array detector. Semi-preparative and analytical C4 or C18 columns with 300 Å pore sizes were purchased from Higgins Analytical. Bulk reversed-phase chromatography was performed on a Biotage system equipped with C4/C18 Sfar Bio 10 g cartridges. Reversed-phase chromatography solvents were as follows: solvent A, 0.1% trifluoroacetic acid (TFA) in H_2_O; solvent B, 0.1% TFA and 90% acetonitrile in H_2_O. Mass spectra were acquired on an Agilent 1290-6545XT liquid chromatography quadrupole time-of-flight (LC-QTOF) electrospray mass spectrometry (ESI–MS) system. All mice were treated in accordance with experimental guidelines approved by either the University of Pennsylvania or the École Polytechnique Fédérale de Lausanne. Data analysis and statistics were performed using GraphPad Prism 8 or 9. Blots and gels for the final main text figures were cropped using Adobe Photoshop 2023.

### Plasmids

A pRK172 construct containing WT human α-syn described previously^54^ was used for the expression of full-length human sequence protein. For experiments requiring the mouse α-syn protein, a custom codon-optimized gene of the mouse sequence was purchased from Integrated DNA Technologies in a pUC19 vector. The mouse sequence was then transferred to the same pRK172 plasmid using 5′ NdeI and 3′ HindIII digestion sites following standard restriction digest cloning protocols. The C-terminal fragment of α-syn (amino acid residues 91–140) was amplified from the full-length construct, then introduced into a pET42b vector using NdeI and Spe I restriction sites. The N-terminal fragment of α-syn (1–84) was also amplified and introduced into a modified pTXB1 construct that contains a C-terminally 6×His-tagged Ava–DnaE N137A intein^34^, using NdeI and Bpu10I restriction sites and standard molecular cloning techniques.

### Expression and purification of full-length human/mouse α-syn

BL21(DE3) competent cells (EMD Millipore) were transformed with pRK172 expression plasmids and selected over ampicillin (100 μg ml^−1^) plates. A single colony was used to inoculate an overnight culture of Luria broth (LB) media grown at 37 °C with shaking at 200 rpm. This overnight culture was expanded 1:100 into terrific broth medium and grown to an optical density (OD) of 0.6–0.8 at 37 °C with shaking. Protein expression was induced by the addition of 0.5 mM isopropyl β-*D*-1-thiogalactopyranoside at room temperature for 16 h. Cells were collected by centrifugation and stored at −20 °C before lysis. Cell pellets were subjected to three rounds of freeze–thaw by submerging in liquid nitrogen for 2 min, followed by thawing in a 37 °C incubator. The pellet was then resuspended in lysis buffer (100 mM Tris, 500 mM sodium chloride (NaCl), 10 mM beta-mercaptoethanol, 1 mM ethylenediamine tetraacetate, pH 8.0) and the resulting slurry was boiled at 80 °C for 10 min. The solution was cooled to room temperature for 30 min, after which protease inhibitor (Roche cOmplete mini, ethylenediamine tetraacetate-free) was added and allowed to incubate for another 30 min on ice. The mixture was clarified by centrifugation, and the pH of the supernatant was slowly adjusted to 3.5. The acidifed mixture was incubated on ice for 30 min and again clarified by centrifugation. The supernatant was dialyzed overnight against degassed 1% acetic acid solution at 4 °C. The dialyzed proteins were then purified via RP-HPLC on a C4 semi-preparative column. Identity and purity were confirmed via analytical liquid chromatography and ESI–MS. The purified protein was lyophilized and stored as dry powder at −20 °C before experiments.

### Expression and purification of recombinant native chemical ligation fragments

For the C-terminal fragment 91–140, protein expression was performed as above for full-length α-syn but using kanamycin as a selection antibiotic for the pET42b plasmid. Lysis and purification were done via the same boiling and acid precipitation protocol described for full-length constructs. After dialysis, the N-terminal cysteine was deprotected via the addition of 100 mM methoxylamine hydrochloride, pH 3.5, at room temperature for 24 h. The cysteine residues were then reduced by adding tris(2-carboxyethyl)phosphine (TCEP) HCl before final purification via C18 semi-preparative RP-HPLC. The identity of the protein fragment was confirmed by ESI–MS. The purified protein was lyophilized and stored as dry powder at −20 °C before chemical ligation.

For the N-terminal fragment 1–84, BL21(DE3) competent cells (EMD Millipore) were transformed with the pTXB1 expression plasmid and selected over ampicillin (100 μg ml^−1^) plates. An overnight culture from a single colony was further expanded 1:100 into fresh terrific broth media at 37 °C with shaking. After reaching an OD of 0.6–0.8, expression was induced with 0.5 mM isopropyl β-*D*-1-thiogalactopyranoside for 16–20 h at room temperature with shaking. Cells were collected by centrifugation and then resuspended in a lysis buffer containing 50 mM sodium phosphate, 300 mM NaCl, 5 mM imidazole and protease inhibitors, pH 7.5. The slurry was sonicated on ice (50% amplitude, 30 s on, 30 s off, 3 min total) and clarified by centrifugation. The supernatant was applied onto prewashed Ni–NTA agarose beads (Qiagen) and incubated at 4 °C with rocking for 1 h. The supernatant was drained, and the beads were washed with 20 column volumes of wash buffer (50 mM sodium phosphate, 300 mM NaCl and 20 mM imidazole, pH 7.5). The intein-fusion protein was eluted with elution buffer (50 mM sodium phosphate, 300 mM NaCl and 250 mM imidazole, pH 7.5). Excess imidazole was removed by dialyzing against PBS at 4 °C overnight. The dialyzed protein solution was clarified by centrifugation, and sodium mercaptoethanesulfonate was added to a final concentration of 200 mM. The pH was adjusted to pH 7, and protein thiolysis was allowed to proceed for 24–48 h. The 1–84 thioester was purified by C4 semi-preparative RP-HPLC, and the identity was confirmed by ESI–MS. The N-terminal thioester protein was stored at −20 °C as lyophilized powder before chemical ligation.

### Solid-phase synthesis of O-GlcNAc α-syn 85–90

Synthesis of the O-GlcNAcylated fragment of α-syn(gS87) was performed via standard, manual fluorenylmethoxycarbony (Fmoc)-based solid-phase methods. Peptides were built on Dawson Dbz AM resin (Novabiochem). Commercially available amino acids (5 equiv.) were activated for 5 min in the presence of hexafluorophosphate benzotriazole tetramethyl uronium (HBTU, 4.5 equiv.) and diisopropylethylamine (10 equiv.) before coupling to the resin for 60 min. Following coupling and washes, N-terminal Fmoc groups were removed with 20% vol/vol piperidine in dimethylformamide (DMF) for 15 min. Deprotection steps were performed twice. O-GlcNAcylated serine residues were coupled using a pentafluorophenyl-activated *per*-acetylated O-GlcNAc Fmoc serine amino acid cassette that was synthesized and purified in house^55^. Two equivalents of the O-GlcNAc amino acid were added to the resin-bound peptide overnight, followed by standard coupling cycles for the remaining amino acids. Residue 85 was coupled as an *N*-Boc-protected thioproline residue. Following completion of the peptide sequence, O-acetyl protecting groups from O-GlcNAc serine residues were removed by the addition of hydrazine hydrate (80% vol/vol in MeOH) twice for 45 min. Before peptide cleavage, the Dawson linker was activated with treatment of *para*-nitrophenyl chloroformate (5 equiv. in CH_2_Cl_2_) for 1 h. Cyclization was effected via incubation with excess *N*,*N*-diisopropylethylamin (DIEA, 5 equiv. in DMF) for 30 min. Peptides were then cleaved from the resin using a standard cleavage cocktail (95:2.5:2.5 TFA/H_2_O/TIPS) for 2 h at room temperature. Crude peptides were precipitated in cold diethyl ether, collected via centrifugation (5,000*g*, 30 min and 4 °C) and lyophilized. This crude material was resuspended in thiolysis buffer (150 mM phosphate and 150 mM sodium mercaptoethanesulfonate pH 6.5) and incubated at room temperature for 2 h before purification via C18 reversed-phase chromatography. Purified peptides were characterized for purity via analytical HPLC, and identity by ESI–MS.

### O-GlcNAc S87 α-syn synthesis

Preparation of O-GlcNAc S87 α-syn was performed as previously published^56^. C-terminal fragment 91–140, and O-GlcNAc S87 peptide 85–90 were incubated in ligation buffer (6 M guanidine HCl, 300 mM phosphate, 25 mM TCEP and 25 mM mercaptophenylacetic acid, pH 7) overnight at room temperature. The protected cysteine in the form of a thiazolidine at residue 85 was deprotected by the addition of 100 mM methoxylamine HCl and adjustment of pH to 3.5. The intermediate corresponding to residues 85–140 was purified by C4 RP-HPLC and lyophilized. A second round of chemical ligation was performed by combining intermediate 85–140 and N-terminal thioester 1–140 in ligation buffer overnight. The product was purified by C4 semi-preparative RP-HPLC and lyophilized. The artificial cysteine residues were then converted to native alanines through radical desulfurization by dissolving the ligation product in degassed buffer containing 6 M guanidine HCl, 300 mM NaCl, 200 mM TCEP HCl, 10% vol/vol *t*-butylthiol, 2% ethanethiol and 2 mM VA-061, pH 7. The desulfurization reaction was stirred over nitrogen at 37 °C for 16 h. The final product was purified via C4 semi-preparative RP-HPLC. Reactions and purity of intermediates and products were monitored by analytical HPLC and ESI–MS.

### Fibrillization from purified monomers

Lyophilized proteins were resuspended in sterile PBS. For Figs. 4–6 and Supplementary Figs. 3–5, Tris-buffered saline (TBS; 50 mM Tris and 150 mM NaCl, pH 7.5) buffer was used. Resuspended proteins were bath sonicated for 15–20 min. Preformed aggregates were removed by centrifugation at 20,000*g* at 4 °C for 20 min. The supernatant was used to determine the protein concentration by standard BCA measurements. The protein concentration was adjusted as indicated in the respective experiments, and the protein solutions were aliquoted into replicate experiments in 1.5 ml microcentrifuge tubes. The tubes were incubated at 37 °C for 5–14 days (as indicated in specific experiments) with shaking at 1,000 rpm in an Eppendorf thermomixer.

### ThT fluorescence

For the kinetic studies in Fig. 2, at indicated time points during the aggregation, 7 μl aliquots were taken from each replicate and stored at −80 °C. At the end of the aggregation period, 5 μl of each time point were thawed and plated onto a 96-well black, clear-bottom microplate. Then, 195 μl of 10 μM ThT in PBS solution were added onto each well. Fluorescence was measured immediately on a Biotek Synergy plate reader using 450 nm excitation, 482 nm emission wavelengths, bottom read and gain setting 100. Data were collected using Gen 5 Version 3.11.19. The fold-increase ThT values reported in the figures were calculated by dividing each measurement by the average of the time 0 baseline ThT measurements.

For routine characterization of the extent of fibrilization, WT and gS87 α-syn fibrils assembled for neuronal and animal studies, assembly solutions were verified by ThT fluorescence, as described previously^57,58^. The sonicated PFFs were resuspended in ThT solution (50 mM glycine pH 8.5 and 10 µM ThT solution), and the ThT fluorescence was measured with a FLUOstar plate reader (BMG Labtech).

### Sedimentation

For the kinetic studies in Fig. 2 and Supplementary Figs 1 and 2, aliquots corresponding to 10 μg of protein were centrifuged at 20,000*g* for 2 h at room temperature. The supernatants were carefully transferred onto new tubes. The same volume of 4% SDS as the original aliquot were added to the pellets, and complete resuspension of the sedimented aggregates was allowed to proceed via bath sonication and boiling for 10 min. SDS–PAGE loading buffer (4×; 4% SDS, 40% glycerol, 0.05% bromophenol blue, 0.252 M Tris–HCl pH 6.8 and 5% β-mercaptoethanol) was added to the supernatant and pellet samples, and these were boiled for an additional 10 min. Equal volumes of supernatant and pellet samples were loaded for each experiment. After SDS–PAGE on 12% Bis–Tris gels and Coomassie staining, soluble and insoluble protein amounts were determined by densitometry using Bio-Rad Image Lab software. Reported percent insoluble values were calculated as the fraction of pellet density over the sum of pellet and soluble densities.

For the analysis of monomer, oligomer and fibril proportions (Extended Data Fig. 3), fibril solutions were analyzed as previously described^57^. The monomeric, oligomeric and fibrillar fractions were resuspended in 4× SDS–PAGE loading buffer 4×, and each fraction was separated on a 1 mm thick 16% Tricine gels for 2 h at 125 V. The proteins in the gel were stained with 0.05% of Coomassie brilliant blue diluted in 25% (vol/vol) isopropanol and 10% acetic acid (vol/vol). The gel was destained with boiling distilled water. As sonication of PFFs can lead to the release of small amounts of monomers, only PFF preparations with residual levels of α-syn monomers lower than 5% were used for the seeding in primary neurons^7^.

### Negative stain EM

The ultrastructures of WT and gS87 α-syn fibrils were analyzed by electron microscopy (EM) as previously described^57^. Activated formvar/carbon-coated 200 mesh copper grids were loaded with 3–5 µl of fibrils sample for 30 s and then washed three times with ultrapure water, before being negatively stained with 1% uranyl acetate for 1–2 min. The excess liquid was removed, and the grids were allowed to air dry. Images were acquired on a FEI Tecnai 12 or Tecnai Spirit BioTWIN electron microscope operating at 80 kV acceleration voltage and equipped with a digital camera (FEI Eagle, FEI).

For analysis of length distribution post-sonication (Extended Data Fig. 3), a total of three to five fields of view for each sample were imaged, and the length of fibrils was quantified using the ImageJ software (US National Institutes of Health; RRID:SCR_001935). Only sonicated PFFs with a 50–100 nm length were used for the seeding in primary neurons^7^.

### PK cleavage

Ten-microgram aliquots of protein were used for each reaction. The indicated amounts of PK were added to each sample to a total volume of 20 μl in Dulbecco’s PBS (DPBS). The reactions were incubated at 37 °C for 30 min. SDS–PAGE sample buffer was added to each reaction, and the samples were boiled for 10 min. The reactions were run on 12% Bis–Tris gels using 2-(*n*-morpholino)ethanesulfonic acid running buffer, and then stained with Coomassie blue.

### Seeded aggregation

Freshly fibrillized unmodified or O-GlcNAc S87 α-syn were subjected to sedimentation. The pellets containing preformed fibers were resuspended in DPBS and the protein concentration determined by BCA. For kinetic studies, the fibril solutions were tip sonicated at 20% amplitude, 1 s on, 1 s off, 20 cycles total. For PK structure templating studies, fibrils were used as is.

Lyophilized unmodified human or mouse α-syn monomers were resuspended in DPBS, bath sonicated for 20 min and centrifuged at 20,000*g* for 20 min at 4 °C. The supernatant was taken and its protein concentration was determined by BCA. Monomers and PFFs were mixed at the indicated ratios, and replicate experiments were prepared in separate tubes. For kinetic experiments, reactions were agitated (1,000 rpm) at 37 °C in an Eppendorf thermomixer for 24 h. Aliquots were taken at the indicated time points and subjected to sedimentation assay immediately, or frozen at −80 °C for later analyses. For templating studies, reactions were agitated for 7 days, then subjected to PK cleavage experiments.

### In vitro PLK3 phosphorylation

Aliquots of α-syn aggregation reactions corresponding to 2.5 μg protein were diluted in reaction buffer (20 mM HEPES, 10 mM magnesium chloride (MgCl_2_) and 2 mM dithiothreitol (DTT), pH 7.4) then subjected to sedimentation. The supernatant was removed to a new tube, and the pellet was resuspended in an equal volume of reaction buffer and then bath sonicated briefly. ATP (NEB, 1 mM final) and protein kinase PLK3 (Thermo PV3812, 2 ng μl^−1^ final concentration) were added to the supernatant or pellet solutions, and the reactions were allowed to proceed for 24 h at 30 °C. The reactions were quenched and solubilized by the addition of 4% SDS and SDS–PAGE loading buffer followed by boiling. The samples were separated via SDS–PAGE and then transferred onto nitrocellulose membrane via semi-dry transfer (Bio-Rad). The membrane was fixed with 4% paraformaldehyde in PBS for 30 min and then washed three times with PBS. Membranes were blocked with OneBlock Western-CL (Genessee Scientific) for 1 h at room temperature. Primary antibodies (Cell Signaling Syn204 mouse mAb or Biolegend 81a 825702 mouse mAb) were added at 1:1,000 dilution and incubated at 4 °C with rocking overnight. The membranes were washed in 1× TBS with Tween (TBST) (Cell Signaling) thrice for 10 min each. Secondary horseradish peroxidase-conjugated antibodies (Jackson Immunoresearch) were added at 1:10,000 dilution and incubated at room temperature for 1 h. The membranes were washed thrice in 1× TBST. Chemiluminescent substrate was added (Bio-Rad Clarity Western ECL), and signal captured on a Bio-Rad ChemiDoc system.

### OGA stability assay

Bacterial OGA (BtGH84) was expressed and purified as previously described^59^. Freshly assembled fibrils or freshly resuspended monomers were diluted in PBS (25 μM final concentration) and mixed with OGA (1 μM final concentration) at a total volume of 50 μl. Reactions were incubated at 37 °C for 4 or 72 h. After the incubation time, reactions were quenched by boiling for 10 min. Reactions were flash frozen in liquid nitrogen and then lyophilized. For analysis, the dried protein was resuspended in 8 M urea and bath sonicated for 5 min. Samples were injected on a C4 analytical RP-HPLC column and monitored on a gradient of 30–60% over 30 min. The identity of the HPLC peaks was confirmed by comparison to runs of protein standards (unmodified or O-GlcNAc S87 protein), as well as analysis by ESI–MS. Quantification was done via integration of the area under the peak using the Agilent Workstation Analysis module.

### In vitro calpain cleavage

Newly assembled fibrils were first subjected to sedimentation. The pellets were then resuspended in calpain reaction buffer (40 mM HEPES, 5 mM DTT and 1 mM CaCl_2_, pH 7.2) and bath sonicated. Calpain (2 U) (EMD 208713) was used per 2.5 μg α-syn as titration experiments showed that this concentration gives about the same amounts of the C-terminal fragment for unmodified or O-GlcNAc S87 fibrils. Reactions were incubated at 37 °C for 30 min and then quenched with 2 mM EGTA. To normalize the concentrations of the truncated fibers for use in seeded aggregation experiments, SDS–PAGE and Coomassie staining were performed to enable densitometric quantitation of the truncated α-syn band (Extended Data Fig. 5).

### Recombinant fibril preparation for neuronal culture experiments and animal injections

For Fig. 3 and Extended Data Fig. 2, after 7 days of assembly from monomers, fibrils were aliquoted and stored at −80 °C. Before use, PFFs were diluted in PBS and sonicated for ten cycles (1 s on, 30 s off, high intensity) in a bath sonicator at 10 °C (BioRuptor Plus; Diagenode).

For Figs. 4–5, Extended Data Figs. 3–4 and Supplementary Figs. 3–5, after 5 days of assembly from monomers, fibrils were fragmented by sonication using a fine probe (20 s, 20% amplitude, 1 pulse on and 1 pulse off) directly after fibril assembly. After sonication, the PFFs were separated from the remaining monomeric solution by following the protocol published by Kumar et al.^57^. The final concentration of the PFFs was quantified by the BCA protein assay according to the supplier’s protocol (Pierce, Thermo Fisher). Sonicated α-syn fibrils were aliquoted and stored at −80 °C.

As previously established^7,57,60^, α-syn fibrils were systematically and thoroughly characterized by sedimentation and the ThT binding assay, in addition to quantitative assessment of the length distribution of the fibrils before and after sonication by EM.

### Neuronal culture condition for pathology and toxicity studies

For experiments in Fig. 3 performed at University of Pennsylvania, primary neuronal cultures were prepared from E16–18 CD1 mouse embryos. Dissociated neurons were plated onto poly-d-lysine-coated optical-bottom 96-well plates (ViewPlate; Perkin Elmer) and 60,000 cells cm^−2^. Cultures were maintained in the Neurobasal medium supplemented with B27 (Invitrogen) that was replenished every 5 days. Sonicated PFFs diluted in sterile PBS without Ca^2+^/Mg^2+^ (Corning) were added at 7 days in vitro (DIV) at the indicated concentrations. Cells were fixed in 4% paraformaldehyde at 19 DIV and co-labeled using antibodies against phosphorylated at serine 129 (mouse monoclonal, clone 81A, 1:10,000; CNDR), NeuN (mouse monoclonal, clone A60; Millipore) and microtubule-associated protein 2 (rabbit, 17028, 1:2,000; CNDR). Alexa Fluor-conjugated secondary antibodies mouse IgG1, IgG2a or rabbit IgG were used to visualize staining. Images were obtained using an InCell2200 scanner (GE Life Sciences).

### Intracerebral injection of PFFs

All housing and procedures were performed according to the National Institutes of Health Guide for the Care and Use of Experimental Animals and approved by the University of Pennsylvania Institutional Animal Care and Use Committee. The injection studies described used 2–3-month-old female B6C3F1/J mice (stock no. 100010; The Jackson Laboratories). Timed-pregnant CD1 mice for neuronal cultures were obtained from Charles River Laboratories. Animals were maintained on a 12 h light/dark schedule and provided with food ad libitum.

Sonicated PFFs (5 μg total in 2.5 μl of PBS) were stereotaxically injected in the dorsal striatum using the following coordinates: AP +0.2 mm, M/L 2.0 mm and depth beneath skull 2.6 mm. Each mouse received a single unilateral injection of the indicated PFFs. At the post-injection time points indicated, mice were transcardially perfused with heparinized PBS, and brains were removed for fixation in 70% ethanol in 150 mM NaCl, pH 7.4.

### Immunohistochemistry

Following fixation, brains were embedded in paraffin and sectioned at 6 µm. Sections were then deparaffinized with in xylene, followed by 1 min washes in a descending series of ethanols (100%, 100%, 95%, 80% and 70%). the slides were then incubated in deionized water for 1 min before antigen retrieval, as noted. After antigen retrieval, slides were incubated in 5% hydrogen peroxide in methanol to quench endogenous peroxidase activity. Slides were then incubated in blocking buffer (0.1 M Tris with 2% fetal bovine serum) and incubated with either antibody against α-syn phosphorylated at serine 129 (mouse monoclonal, clone 81A, 1:10,000), misfolded α-syn (mouse monoclonal, clone Syn506, 1:2,000) or tyrosine–hydroxylase (mouse monoclonal; clone TH-16, 1:1,000; Sigma). Primary antibody was rinsed off with 0.1 M Tris three times for 5 min, then incubated with biotinylated horse anti-mouse IgG (1:1,000; Vector BA2000) in blocking buffer for 1 h. Sections were then washed and incubated with avidin–biotin solution (Vector PK-6100) for 1 h. Slides were then washed and developed with ImmPACT DAB peroxidase substrate (Vector SK-4105) and counterstained briefly with hematoxylin. Slides were rinsed in water, dehydrated and coverslipped in Cytoseal (Fisher 23-244-256). All slides were digitized using a Lamina Scanner (Perkin Elmer) in brightfield mode.

### Preparation and treatment of WT and α-syn KO hippocampal primary neurons

For experiments in Figs. 4 and 5, Extended Data Figs. 4 and 5 and Supplementary Figs 3–5 performed at EPFL, WT and α-syn KO primary hippocampal neurons were respectively prepared from WT C57BL/6JRj (Janvier) or α-syn KO (C57BL/6J OlaHsd, Envigo) pups at postnatal day 0 (P0) as previously reported^61^. The dissociated neurons were plated in 6-well plates, onto coverslips (VWR) or in clear black-bottom 96-well plates (Falcon) freshly coated with poly-l-lysine 0.1% wt/vol in water (Brunschwig). Neurons were plated at a density of 300,000 cells per ml for biochemistry analyses, 250,000 cells per ml for the ICC analyses and 200,000 cells per ml for the high-content imaging analysis (HCA). The α-syn KO neurons were treated after 10 days in culture (DIV 13) with extracellular human WT or gS87 α-syn PFFs at a final concentration of 70 nM. The uptake, proteolytic processing and clearance of the PFFs were followed from 14 h after the addition of the seeds and up to 10 days. In WT hippocampal primary neurons, the WT or gS87 α-syn PFFs seeds were added at DIV 6 or DIV 13 at a final concentration of 70 nM and the PFF-treated neurons were all fixed or lysed at DIV 27 as previously described^5–7,62^. PBS was used as a negative control. All procedures were approved by the Swiss Federal Veterinary Office (authorization number VD3496).

### Assessment of α-syn PFF biochemistry and seeding activity in α-syn KO and WT hippocampal primary neurons

PBS- or PFFs-treated α-syn KO and WT neurons were lysed in 1% Triton X-100/TBS (50 mM Tris and 150 mM NaCl, pH 7.5) supplemented with 1/100 of protease inhibitor cocktail (Sigma-Aldrich), 1 mM PMSF (Sigma-Aldrich) and 1/100 of phosphatase inhibitor cocktails 2 and 3 (Sigma-Aldrich). The cell lysates were sonicated with a fine probe (0.5 s pulse at 20% amplitude, ten times) and then incubated on ice for 30 min and centrifuged at 100,000*g* for 30 min at 4 °C. The supernatant (soluble fraction) was collected in a new tube. The pellet was washed in 1% Triton X-100/TBS and then sonicated for (1 s pulse on, 1 s pulse off at 20% amplitude, ten times) and centrifuged for another 30 min at 100,000*g*. The supernatant was discarded, and the pellet (insoluble fraction) was resuspended in 2% SDS/TBS supplemented with 1/100 of protease inhibitor cocktail, 1 mM PMSF and 1/100 of phosphatase inhibitor cocktails 2 and 3. The pellet was sonicated using the fine probe (1 s pulse on, 1 s pulse off at 20% amplitude, 15 times). The protein concentration in the soluble and insoluble fractions was quantified by the BCA protein assay according to the supplier’s protocol (Pierce, Thermo Fisher). The soluble and insoluble fractions were next resuspended in Laemmli buffer 4× (4% SDS, 40% glycerol, 0.05% bromophenol blue, 0.252 M Tris–HCl pH 6.8 and 5% β-mercaptoethanol). The proteins from each fraction were separated on 1-mM-thick 16% Tricine gels (Life Technologies) for 2 h at 125 V. The proteins were then transferred onto nitrocellulose membranes (0,2 µm, GE Healthcare) using a semi-dry transfer system (Life Technologies) under a constant current (25 V) and a maximum tension of 0.5 A. The membranes were then blocked for 1 h at room temperature in Odyssey blocking buffer (Li-COR Biosciences) and probed with the relevant primary antibodies (Supplementary Table 1) overnight at 4 °C. After three washes with PBS buffer containing 0.1% (vol/vol) Tween 20 (Fluka) (PBS-T), the membranes were incubated with secondary goat anti-mouse or anti-rabbit antibodies conjugated to Alexa fluor 680 or 800 dyes (Li-COR Biosciences). The membranes were then washed three times with PBS-T, and scanned on a Li-COR scanner (Li-COR Biosciences). The level of total α-syn or pS129 α-syn was estimated by measuring the WB band intensity using Image Studio software (Li-COR Biosciences, RRID*:*SCR_015795) and normalized to the relative protein levels of actin. All the experiments were independently repeated three times.

### ICC

PBS- or PFFs-treated hippocampal primary neurons were fixed in 4% formaldehyde (Sigma-Aldrich) for 20 min at room temperature and immunostained. The PFFs were detected using a total α-syn antibody (SYN1). The seeded aggregates were detected using the mouse monoclonal pS129 (81a, Biolegend) or the rabbit monoclonal pS129 (MJFR-13, Abcam) antibodies and were costained with the ubiquitin or the p62 antibodies or with the Amytracker dye (Ebba Biotech). LAMP1 antibody was used to stain the late endosome/lysosomes. The neurons were counterstained with the microtubule-associated protein (MAP2) antibody and the nucleus with DAPI staining. All the information about the antibodies used in this study can be found in Supplementary Table 1. PBS- or PFFs-treated neurons plated on coverslips were imaged with a confocal laser-scanning microscope (LSM 700, Carl Zeiss Microscopy) with a 40× objective and analyzed using the Zen software (Carl Zeiss Microscopy, RRID:SCR_013672). The PBS- or PFFs-treated neurons plated in black clear-bottom 96-well plates were imaged using the IN Cell Analyzer 2200 plate reader as previously described^6,7^. For each independent experiment, three wells were acquired per condition, and in each well, nine fields of view were imaged. Each independent experiment was reproduced at least three times. The identification of the pS129-positive seeded aggregated formed in neurons MAP2-positive neuronal cells and the quantification of the pS129 intensity was performed using Cell profiler 3.0.0 software (RRID:SCR_007358) as previously described^7^.

### Statistics

The data from at least three independent experiments were analyzed. The statistical analyses were performed using one-way analysis of variance (ANOVA) followed by the multicomparison Tukey’s honest significant difference post hoc test (compared groups are specified in the corresponding legend). The data were regarded as statistically significant at *P* < 0.05.

### HSP27 fibril seeding inhibition assay

Purified HSP27 (200 µg) C137A (prepared as previously described^50^) was denatured in 6 M GnHCl, 50 mM sodium phosphate pH 8 buffer and dialyzed overnight at 4 °C into DPBS for slow refolding. The protein was concentrated using 10 kDa MWCO Amicon Ultra-0.5 Centrifugal Filters and quantified by BCA. Monomeric α-syn was prepared by resuspending the lyophilized protein in PBS buffer and bath sonicating for 15 min, after which the solution was clarified by centrifugation at 20,000*g* for 30 min. The solution was then spin filtered through Microcon DNA fast flow Ultracel regenerated cellulose columns to remove oligomers for 20 min. The α-syn monomer and ThT stock solution were combined to a concentration of 62.5 µM each in PBS. This monomeric α-syn solution was divided into three conditions for treatment with different concentrations of HSP27. After incubation of the monomer–HSP mixtures at 37 °C for 30 min, seeds were added to 5% final volume for a final concentration of 50 µM monomer, and 50 µM ThT, 2.5 µM preformed fibers and respective HSP27 concentration (no HSP27, 0.125 µM HSP27 and 0.5 µM HSP27).

Then, 120 µl of each solution was pipetted into the wells of a clear-bottomed black 96-well plate. Before the assay, the BioTek Cytation 5 instrument was preheated to 37 °C. The excitation wavelength was set to 450 nm, and emission to 482 nm for ThT fluorescence monitoring over the course of 24 h, with readings taken every 5 min without shaking. The experiments were done in triplicate.

### Cryo-EM sample preparation and data collection

The cryo-EM grid preparation was performed at the Core Facilities of the Structural Biology Laboratory at the University of Texas Southwestern Medical Center (UTSW) using a Vitrobot Mark IV (FEI). A 3 μl aliquot of fibril sample (assembled over 14 days) was pipetted on a glow-discharged Quantifoil R 1.2/1.3, 300 mesh, Cu grid. The excess sample was removed by blotting and the grid was then plunged into liquid ethane. The cryo-EM samples were screened on either the Talos Arctica or Glacios at the Cryo-Electron Microscopy Facility at UTSW. The cryo-EM data were acquired using a TEM Beta, a 300 kV Titan Krios G3i electron microscope equipped with a K3 detector, at the Stanford–SLAC Cryo-EM Center (S2C2). The pixel size, frame rate, dose rate, final dose and number of micrographs are described in detail in Supplementary Information. The data collection was automated using the EPU 2.8 software package.

### Data preprocessing

All data preprocessing and processing steps were performed using RELION 3.1, unless specified otherwise^63^. The raw movie frames were gain corrected, aligned, motion corrected and dose weighted using the motion correction program implemented in RELION^64^. Contrast transfer function was estimated using CTFFIND 4.1 (ref. ^65^). The filaments were manually picked without discrimination using EMAN2 e2helixboxer.py^66^.

### Helical reconstruction

The filaments were extracted using 1,024 pixel boxes with a 10% inter-box distance and downscaled to 256 pixels. Two-dimensional classification was performed to select the most visually appealing classes and generate a de novo 3D initial model using the relion_helix_inimodel2d script^67^. The fibril helix was assumed to have a left-handed orientation for 3D reconstruction. Several rounds of 3D classification were carried out to determine the optimal reconstructed class. The segments from the selected class were re-extracted in 320 pixel boxes without further downscaling and underwent another round of 3D classification with an initial regularization parameter (T factor) of 4 and a large angular sampling interval of 7.5°. The T factor was gradually increased while the angular sampling was decreased stepwise with close manual monitoring of the reconstructed results. The helical twist and rise were optimized once the estimated resolution of the model exceeded 4.75 Å. After reaching a T factor of 128 and an angular sampling of 1.8, the T factor was slowly decreased to 8 with an angular sampling of 1.8 for the final map of this step. This map underwent 3D auto-refinement, then post-processed using a 10 pixel extended initial binary mask. The final resolution was estimated using the 0.143 threshold FSC between two independently refined half-maps^68^.

### Model building and refinement

The final 3D classification map was used for model building, as the resolution of the resulting map from 3D auto-refine was low, resolution 5.0. The model was built in COOT using the residue sequence V77–A89 from the recombinant α-syn structure (Protein Data Bank (PDB) code 6A6B) as the template^67^. To improve the model’s accuracy, multiple rounds of refinement were performed with the default settings in phenix.real_space_refine with noncrystallographic symmetry constraints, minimization_global, rigid_body and local_grid_search^69^. The model’s geometry was assessed using MolProbity, a tool integrated in Phenix. In between each refinement round, regions of the model with problematic fit or low quality were manually adjusted using COOT. This process was repeated until the model exhibited an acceptable level of stereochemistry and an adequate overall correlation coefficient with the map.

### Reporting summary

Further information on research design is available in the Nature Portfolio Reporting Summary linked to this article.

## Online content

Any methods, additional references, Nature Portfolio reporting summaries, source data, extended data, supplementary information, acknowledgements, peer review information; details of author contributions and competing interests; and statements of data and code availability are available at 10.1038/s41589-024-01551-2.

### Supplementary information

Supplementary Information Supplementary Figs. 1–5, Table 1 and cryo-EM data.

Reporting Summary

### Source data

Source Data Fig. 2 Unprocessed blots.

Source Data Fig. 2 Statistical source data.

Source Data Fig. 3 Statistical source data.

Source Data Fig. 4 Statistical source data.

Source Data Fig. 5 Statistical source data.

Source Data Extended Data Fig. 2 Statistical source data.

Source Data Extended Data Fig. 6 Statistical source data.

## Data Availability

All data generated or analyzed during this study are included in this published article and the accompanying files. Plasmids encoding α-syn protein constructs used in this study are freely available upon request. The structure of α-syn(gS87) 8GF7 is deposited in the PDB at https://www.rcsb.org/. Source data are provided with this paper. Full, uncropped blots are available in Supplementary Information or Source data.
