# Author Correction: O-GlcNAc forces an α-synuclein amyloid strain with notably diminished seeding and pathology

**DOI:** 10.1038/s41589-024-01587-4

**Published:** 2024-02-27

**Authors:** Aaron T. Balana, Anne-Laure Mahul-Mellier, Binh A. Nguyen, Mian Horvath, Afraah Javed, Eldon R. Hard, Yllza Jasiqi, Preeti Singh, Shumaila Afrin, Rose Pedretti, Virender Singh, Virginia M.-Y. Lee, Kelvin C. Luk, Lorena Saelices, Hilal A. Lashuel, Matthew R. Pratt

**Affiliations:** 1 Department of Chemistry, University of Southern California, Los Angeles, CA USA; 2 Laboratory of Molecular and Chemical Biology of Neurodegeneration, Institute of Bioengineering, School of Life Sciences, École Polytechnique Fédérale de Lausanne, Lausanne, Switzerland; 3 Center for Alzheimer’s and Neurodegenerative Diseases, Department of Biophysics, Peter O’Donnell Jr. Brain Institute, UT Southwestern Medical Center, Dallas, TX USA; 4 The Department of Pathology and Laboratory Medicine, Institute on Aging and Center for Neurodegenerative Disease Research, the Perelman School of Medicine, University of Pennsylvania, Philadelphia, PA USA; 5 Department Biological Sciences, University of Southern California, Los Angeles, CA USA

**Keywords:** Glycobiology, Protein aggregation, Post-translational modifications, Proteins

Correction to: *Nature Chemical Biology* 10.1038/s41589-024-01551-2, published online 12 February 2024.

In the version of the article initially published, in Fig. 5c, the western blot data for the actin loading in the soluble protein fraction was inadvertently a reproduction of the actin loading from the insoluble protein fraction. The correct actin loading was shown in the middle panel of Supplementary Fig. 5 in the original publication. Fig. 5c has been replaced in the HTML and PDF versions of the article to reflect the correct data.

