# Supplementary material for: O-GlcNAc forces an α-synuclein amyloid strain with notably diminished seeding and pathology
Source: Nat Chem Biol. Author manuscript; Available in PMC 2025 May 1. (PMC11062923; doi:10.1038/s41589-024-01551-2)
Supplement: Supplementary Information [file NIHMS1970379-supplement-Supplementary_Information.pdf]

nature chemical biology

Article https://doi.org/10.1038/s41589-024-01551-2

O-GlcNAc forces an α-synuclein amyloid strain with notably diminished seeding and pathology

In the format provided by the authors and unedited

O-GlcNAc modification forces the formation of an α-Synuclein amyloid-strain with notably diminished seeding activity and pathology

Aaron T. Balana,[1#] Anne-Laure Mahul-Mellier,[2#] Binh A Nguyen,[3] Mian Horvath,[4] Afraah Javed,[1] Eldon R. Hard,[1] Yllza Jasiqi,[2] Preeti Singh,[3] Shumaila Afrin,[3] Rose Pedretti,[3] Virender Singh,[3] Virginia M.-Y. Lee,[4] Kelvin C. Luk,[4] Lorena Saelices Gomez,[3] Hilal A. Lashuel,[2,*] and Matthew R. Pratt[1,5,*]

[1]Departments of Chemistry and [5]Biological Sciences, University of Southern California, Los Angeles, CA 90089, United States
[2]Laboratory of Molecular and Chemical Biology of Neurodegeneration, Institute of Bioengineering, School of Life Sciences, École Polytechnique Fédérale de Lausanne, Lausanne, Switzerland CH-1015
[3]Center for Alzheimer's and Neurodegenerative Disease, Department of Biophysics, Peter O'Donnell Jr. Brain Institute, UT Southwestern Medical Center, Dallas, TX-75390
[4]The Department of Pathology and Laboratory Medicine, Institute on Aging and Center for Neurodegenerative Disease Research, the Perelman School of Medicine, University of Pennsylvania, Philadelphia, PA, USA.

#These authors contributed equally

*Corresponding authors: Hilal A. Lashuel, hilal.lashuel@epfl.ch; Matthew R. Pratt, matthew.pratt@usc.edu

Table of contents:

Supplementary Figure 1. Full size blots of seeded aggregation of human α-Syn. **Page S2**
Supplementary Figure 2. Full size blots of seeded aggregation of mouse α-Syn. **Page S3**
Supplementary Figure 3. Unmodified and α-Syn(gS87) PFFs display similar processing in neurons. **Page S4**
Supplementary Figure 4. Unmodified and α-Syn(gS87) PFFs display similar stability in neurons. **Page S5**
Supplementary Figure 5. α-Syn(gS87) PFFs have dramatically reduced seeding capacity in neurons. **Page S5**
Supplementary Table 1. Antibodies used in this study **Page S6**
Cryo-EM data collection, reconstruction, model building, and validation. **Page S7**

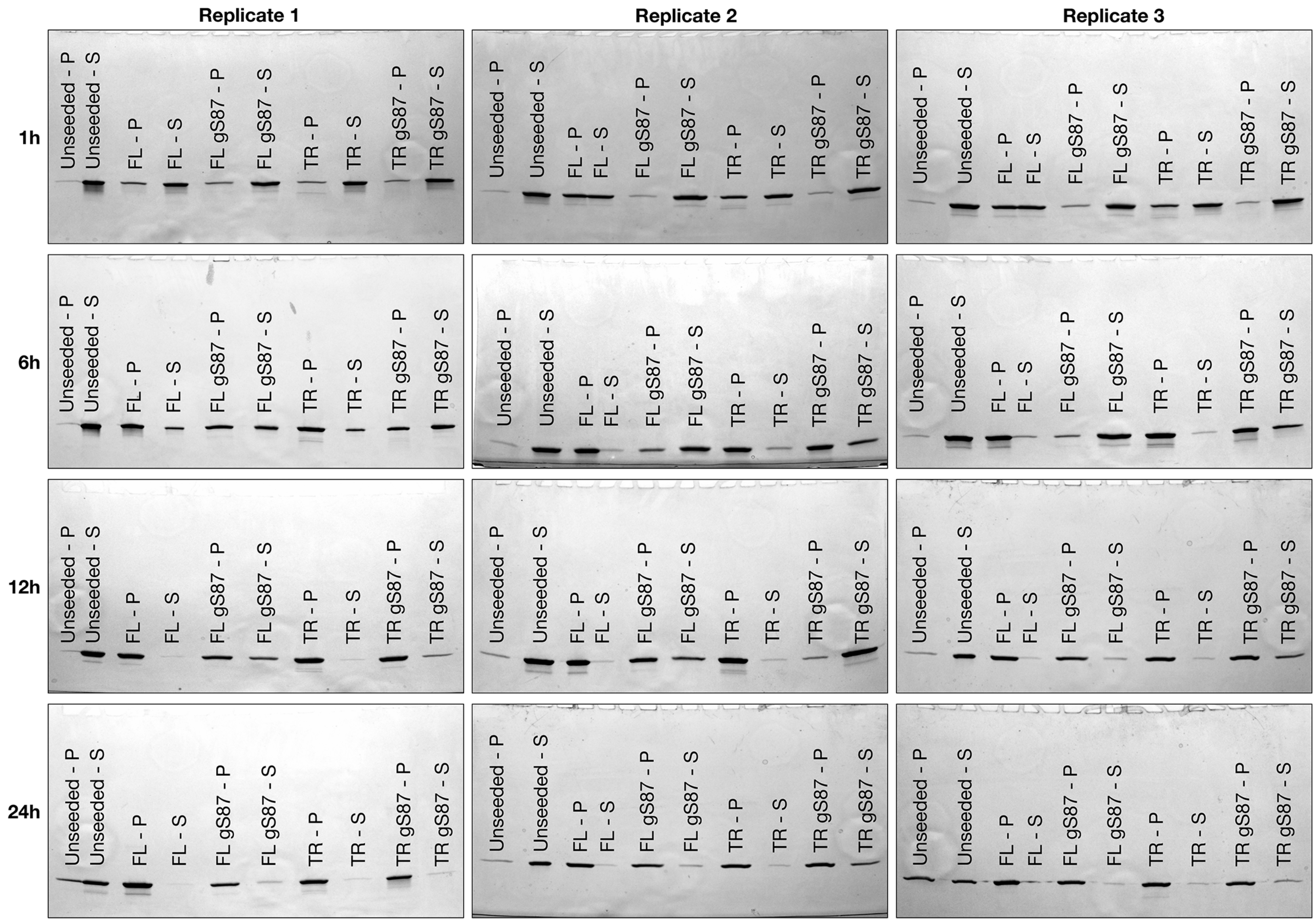

Supplementary Figure 1. Full size blots of seeded aggregation of human α-Syn. Raw data for Figures 2f and 6a. FL = full length α-Syn, FL gS87 = full length α-Syn(gS87), TR = truncated α-Syn, TR gS87 = truncated α-Syn(gS87), P = pellet, S = soluble.

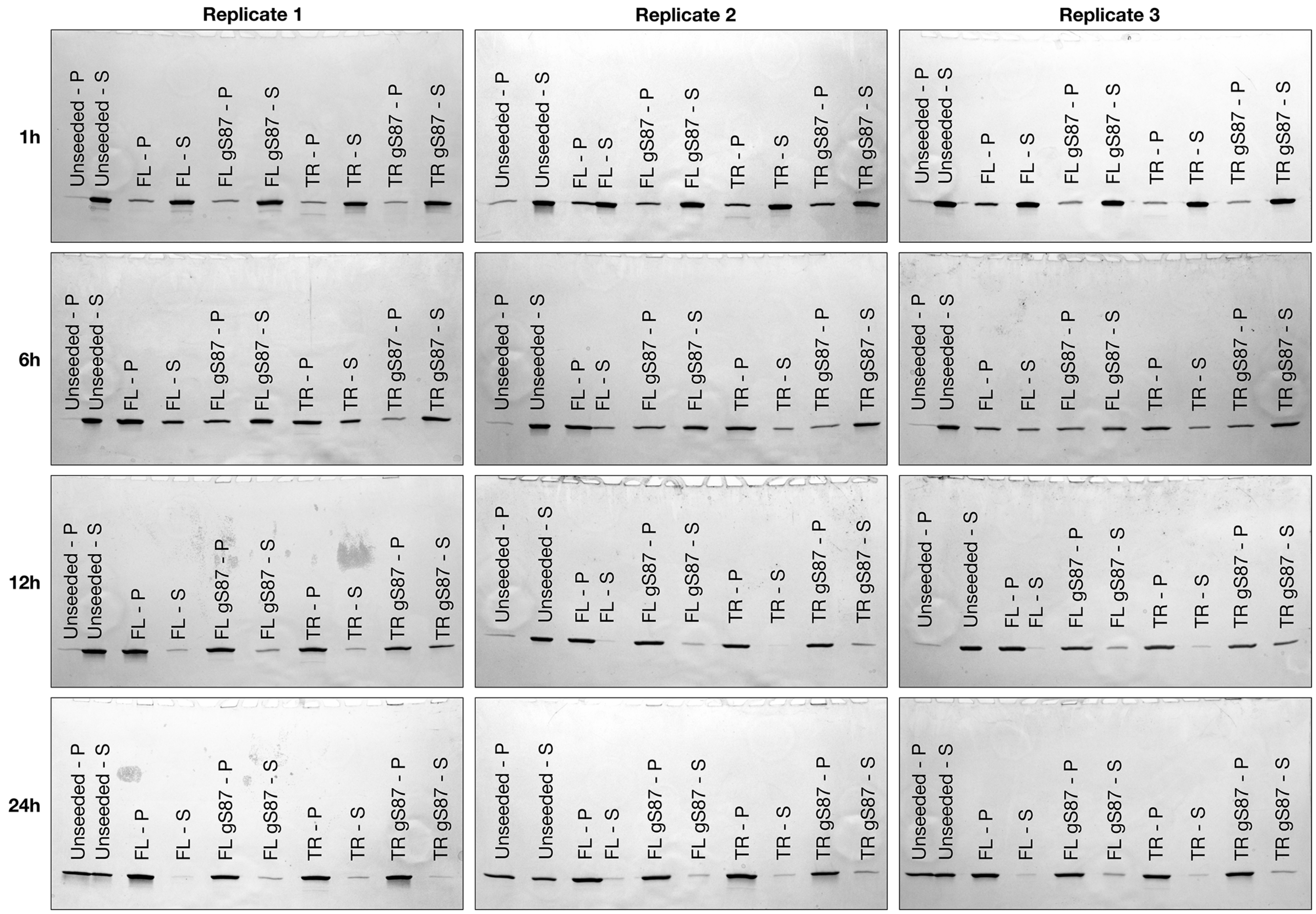

Supplementary Figure 2. Full size blots of seeded aggregation of mouse α-Syn. Raw data for Figures 2h and 6b. FL = full length α-Syn, FL gS87 = full length α-Syn(gS87), TR = truncated α-Syn, TR gS87 = truncated α-Syn(gS87), P = pellet, S = soluble.

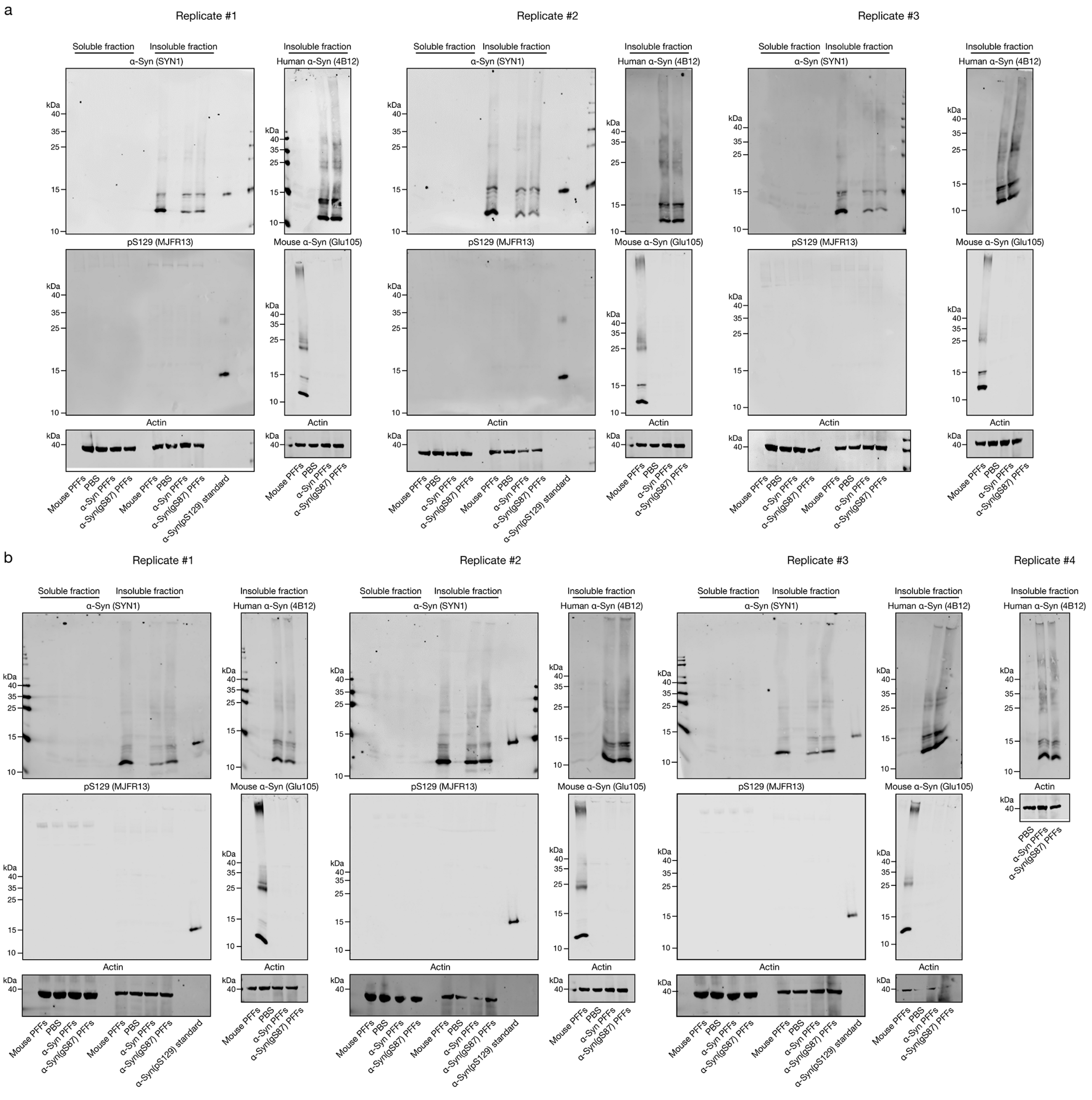

Supplementary Figure 3. Unmodified and α-Syn(gS87) PFFs display similar processing in neurons. Primary hippocamal neurons from α-synuclein knockout-mice at 7 days in vitro (7 DIV) were treated with the indicated human or mouse PFFs (70 nM) or PBS for different lengths of time before the following analyses. a) O-GlcNAc at S87 largely does not affect the the internalization, C-terminal cleavage to ~12 kDa fragment, or phosphorylation at S129 (pS129) of PFFs as visualized by western blotting after 14 h of treatment. b) O-GlcNAc at S87 largely does not affect the the internalization, C-terminal cleavage to ~12 kDa fragment, or phosphorylation at S129 (pS129) of PFFs as visualized by western blotting after 24 h of treatment.

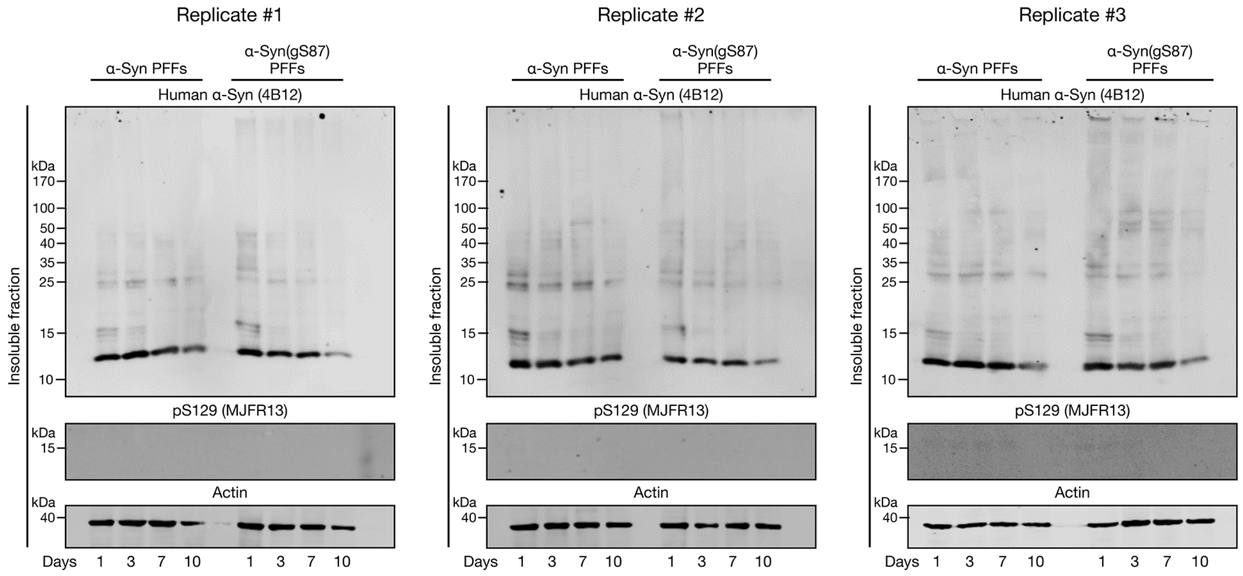

Supplementary Figure 4. Unmodified and α-Syn(gS87) PFFs display similar stability in neurons. Primary hippocamal neurons from α-synuclein knockout-mice at 7 days in vitro (7 DIV) were treated with the indicated human PFFs (70 nM) or PBS for different lengths of time before visualization by western blotting over 10 days of treatment.

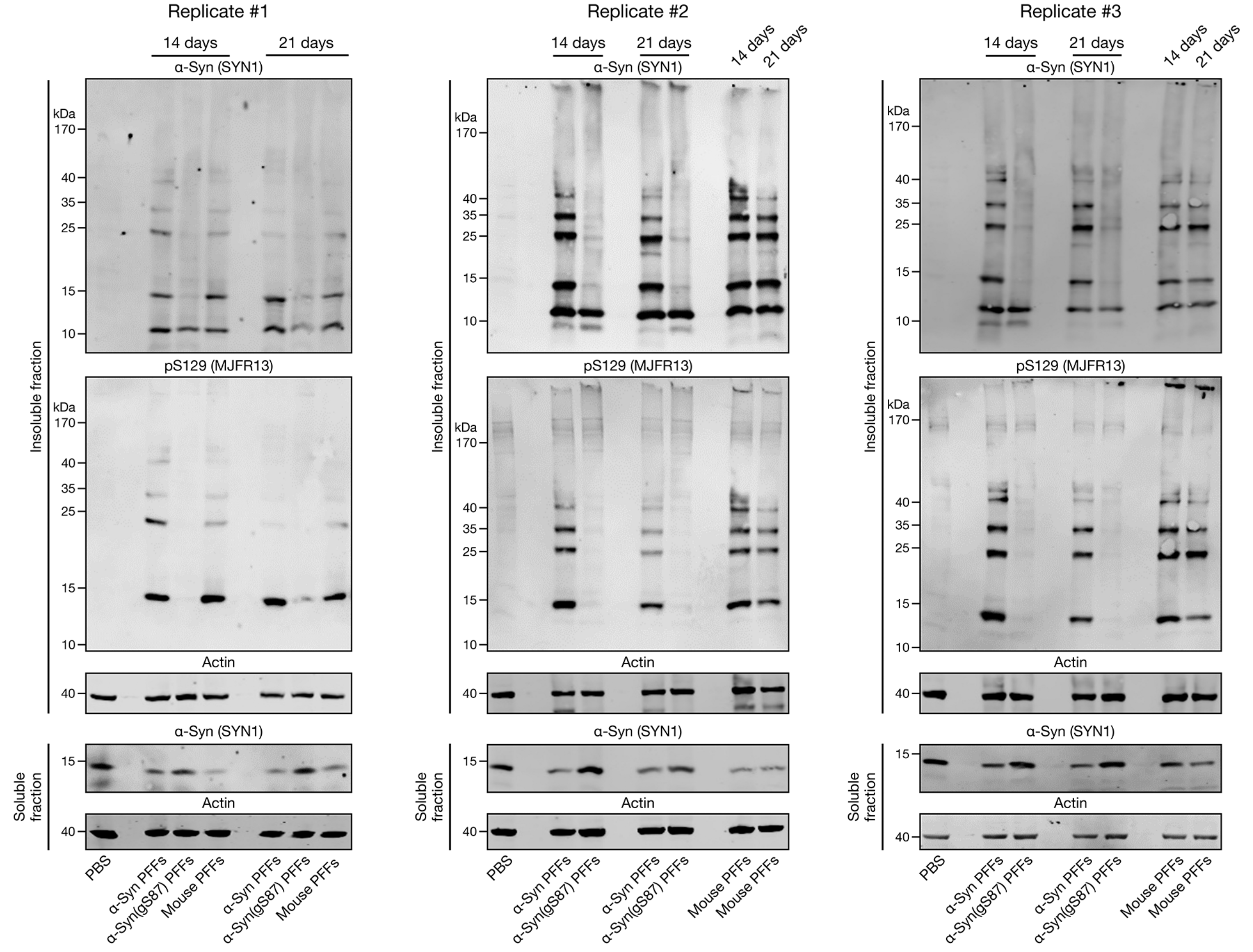

Supplementary Figure 5. α-Syn(gS87) PFFs have dramatically reduced seeding capacity in neurons. Primary hippocampal neurons from wild-type mice at 7 days in vitro (7 DIV) were treated with the indicated PFFs (70 nM) or PBS for different lengths of time before analysis by western blotting. Unmodified PFFs seed the aggregation of endogenous α-synuclein into insoluble and pS129-positive higher molecular-weight aggregates. O-GlcNAc at S87 dramatically reduced this seeded aggregation and more endogenous α-synuclein remained soluble.

Supplementary Table 1. Antibodies used in this study.

Primary Antibody	Catalog #	Company	Clone	RRID	Host	Concentration	WB dilution	IC dilution	Epitope
anti-α-Syn total	2647	Cell Signaling	Syn204	AB_2302251	Mouse	0.2 mg/mL	1 :1000	-	
anti-α-Syn total	610787	BD	SYN-1	AB_398108	Mouse	0.25 mg/mL	1:1000	1:1000	91-99
anti-α-Syn misfolded	-	CNDR	Syn506	NA	Mouse	1.0 mg/mL	-	1 :2000	Conformational epitope associated with residues 1-12
anti-pS129-α-Syn	825701	BioLegend	p-syn /81A	AB_2564891	Mouse	1.0 mg/mL	1:1000	1:2000	Peptide (residues 124-134) including phosphorylated Ser129 of human α-Syn
anti-pS129-α-Syn (Luk lab)	-	CNDR	81A	NA	Mouse	3 mg/mL	-	1 :10,000	Peptide (residues 124-134) including phosphorylated Ser129 of human α-Syn
anti-pS129-α-Syn	ab168381	Abcam	MJF-R13	AB_2728613	Rabbit	4.229 mg/mL	1:3000	1:3000	The exact sequence is proprietary
anti-NeuN	MAB377	Millipore	A60	AB_2298772	Mouse	1 mg/mL	-	1 :2000	
anti-TH	T2928	Sigma	TH-16	AB_2313844	Mouse	5 mg/mL	-	1 :1000	
anti-LAMP1	ab24170	Abcam	-	AB_775978	Rabbit	1.0mg/mL	-	1:1000	
anti-p62	H00008878	Abnova	2C11	AB_437085	Mouse	1 mg/ml	1:1000	1:500	raised against a full length recombinant SQSTM1
anti-ubiquitin	Sc-8017	Santa-Cruz	P4D1	AB_628423	Mouse	0.2 mg/ml	1:500	1:500	1-76
anti-Actin	ab6276	Abcam	AC-15	AB_2223210	Mouse	2.2 mg/mL	1:5000	-	
anti-MAP2	-	CNDR	17028	NA	Rabbit	Not provided	-	1 :2000	
anti-MAP2	ab92434	Abcam	-	AB_2138147	Chicken	Not provided	Not tested	1:2000	

Secondary Antibody	Catalog #	Company	RRID	Concentration	WB dilution	IC dilution
Donkey anti-mouse peroxidase	715-035-150	Jackson Immunoresearch	AB_2340770	0.8 mg/mL	1 :10,000	-
Horse anti-mouse biotinylated	BA2000	Vector	AB_2313581	1.5 mg/mL	-	1 :1000
Goat anti-mouse Alexa Fluor 680	A21058	Invitrogen	AB_2535724	2 mg/ml	1:5000	-
Goat anti-rabbit Alexa Fluor 800	926-32211	Li-Cor	AB_621843	1 mg/ml	1:5000	-
Donkey anti-rabbit Alexa Fluor 647	A31573	Invitrogen	AB_2536183	2 mg/ml	-	1:800
Donkey anti-mouse Alexa Fluor 647	A31571	Invitrogen	AB_162542	2 mg/ml	-	1:800
Goat anti-chicken Alexa Fluor 568	A11041	Invitrogen	AB_2534098	2 mg/ml	-	1:500
Goat anti-mouse Alexa Fluor 488	A-11029	Invitrogen	AB_2534088	2 mg/ml	-	1:800
Donkey anti-chicken Alexa Fluor 488	703-545-155	Jackson Immunoresearch	AB_2340375	1 mg/ml	-	1:400
Donkey anti-rabbit Alexa Fluor 488	A21206	Invitrogen	AB_2535792	2 mg/ml	-	1:800
Amytracker 680	-	EBBA Biotech	Not available	1 mg/ml	-	1:100

Cryo-EM data collection, refinement and validation statistics

	α-Syn(gS87) (EMDB-29980) (PDB 8GF7)
**Data collection and processing**	
Magnification	105,000
Voltage (kV)	300
Electron exposure (e–/Å^2)	62
Defocus range (μm)	-0.8 to -2.2
Pixel size (Å)	0.86
Symmetry imposed	C1
Initial particle images (no.)	120526
Final particle images (no.)	10410
Map resolution (Å)	4.3
FSC threshold	0.143
Map resolution range (Å)	4.0 – 5.8
**Refinement**	
Initial model used (PDB code)	6A6B
Model resolution (Å)	4.1
FSC threshold	0.143
Model resolution range (Å)	n/a
Map sharpening *B* factor (Å^2)	134
Model composition	
Non-hydrogen atoms	3690
Protein residues	540
Ligands	0
B factors (Å^2)	
Protein	125
Ligand	0
R.m.s. deviations	
Bond lengths (Å)	0.002
Bond angles (°)	0.585
Validation	
MolProbity score	2.13
Clashscore	11.45
Poor rotamers (%)	0
Ramachandran plot	
Favored (%)	89.77
Allowed (%)	10.23
Disallowed (%)	0
