# Supplementary material for: O-GlcNAc forces an α-synuclein amyloid strain with notably diminished seeding and pathology
Source: Nat Chem Biol. 2024 Feb 12;20(5):646–55. doi: 10.1038/s41589-024-01551-2 (PMC11062923; doi:10.1038/s41589-024-01551-2)
Supplement: Supplementary file 2 [file 41589_2024_1551_MOESM2_ESM.pdf]

nature portfolio

Corresponding author(s): Matthew Pratt and Hilal Lashuel

Last updated by author(s): Nov 8, 2023

Reporting Summary

Nature Portfolio wishes to improve the reproducibility of the work that we publish. This form provides structure for consistency and transparency in reporting. For further information on Nature Portfolio policies, see our Editorial Policies and the Editorial Policy Checklist.

Statistics

For all statistical analyses, confirm that the following items are present in the figure legend, table legend, main text, or Methods section.

n/a	Confirmed	
☐	☒	The exact sample size (*n*) for each experimental group/condition, given as a discrete number and unit of measurement
☐	☒	A statement on whether measurements were taken from distinct samples or whether the same sample was measured repeatedly
☐	☒	The statistical test(s) used AND whether they are one- or two-sided<br>*Only common tests should be described solely by name; describe more complex techniques in the Methods section.*
☒	☐	A description of all covariates tested
☒	☐	A description of any assumptions or corrections, such as tests of normality and adjustment for multiple comparisons
☐	☒	A full description of the statistical parameters including central tendency (e.g. means) or other basic estimates (e.g. regression coefficient) AND variation (e.g. standard deviation) or associated estimates of uncertainty (e.g. confidence intervals)
☐	☒	For null hypothesis testing, the test statistic (e.g. *F*, *t*, *r*) with confidence intervals, effect sizes, degrees of freedom and *P* value noted<br>*Give P values as exact values whenever suitable.*
☒	☐	For Bayesian analysis, information on the choice of priors and Markov chain Monte Carlo settings
☒	☐	For hierarchical and complex designs, identification of the appropriate level for tests and full reporting of outcomes
☒	☐	Estimates of effect sizes (e.g. Cohen's *d*, Pearson's *r*), indicating how they were calculated

Our web collection on statistics for biologists contains articles on many of the points above.

Software and code

Policy information about availability of computer code

Data collection	BioTek Gen5 Version 3.11.19, Image Studio software (Li-COR Biosciences, RRID:SCR_015795), I, InCell2200 scanner software (GE Life Sciences), Lamina Scanner Software (Perkin Elmer), EPU 2.8 software package.
Data analysis	Photoshop 2023, BioRad Image lab 4.1, Graphpad Prism 8 & 9, BioRad Image lab 4.1, Cell profiler 3.0.0, RELION 3.1, COOT, CTFFIND4.1, EMAN2 e2helixboxer.py, MolProbity (Phenix)

For manuscripts utilizing custom algorithms or software that are central to the research but not yet described in published literature, software must be made available to editors and reviewers. We strongly encourage code deposition in a community repository (e.g. GitHub). See the Nature Portfolio guidelines for submitting code & software for further information.

Data

Policy information about availability of data

All manuscripts must include a data availability statement. This statement should provide the following information, where applicable:

- Accession codes, unique identifiers, or web links for publicly available datasets
- A description of any restrictions on data availability
- For clinical datasets or third party data, please ensure that the statement adheres to our policy

All data generated or analyzed during this study are included in this published article and the accompanying files. Plasmids encoding α-Syn protein constructs used

in this study are freely available upon request. The structure of α-Syn(gS87) 8GF7 is deposited in the Protein Data Bank (PDB) at https://www.rcsb.org/. Full, uncropped blots are available in the Supporting Information.

Human research participants

Policy information about studies involving human research participants and Sex and Gender in Research.

Reporting on sex and gender	N/A
Population characteristics	N/A
Recruitment	N/A
Ethics oversight	N/A

Note that full information on the approval of the study protocol must also be provided in the manuscript.

Field-specific reporting

Please select the one below that is the best fit for your research. If you are not sure, read the appropriate sections before making your selection.

☒ Life sciences ☐ Behavioural & social sciences ☐ Ecological, evolutionary & environmental sciences

For a reference copy of the document with all sections, see nature.com/documents/nr-reporting-summary-flat.pdf

Life sciences study design

All studies must disclose on these points even when the disclosure is negative.

Sample size	We expected that cohorts of 5 mice/ treatment arm will have 0.8 power to detect a 30% mean difference for a continuous outcome (e.g. abundance of aSyn inclusions, cell loss) between two groups/hemispheres at the 0.05 significance level. Based on our previous work, we anticipate the effect size to be considerably larger, hence providing sufficient power for our in vivo studies.
Data exclusions	No data points were excluded in any of the analysis
Replication	All experiments were repeated as separate experiments at least once, and all attempts at replication were successful.
Randomization	For all experiments cells were randomly split into wells for the different experimental conditions. Injected mice were selected randomly and each served as both control and experiment through injection into one brain hemisphere.
Blinding	We did not blind the majority of the biochemical and cell culture experiments as they produce readily quantifiable data. For animal studies, analysis was performed with the experimenters blinded to the treatment group of each animal.

Reporting for specific materials, systems and methods

We require information from authors about some types of materials, experimental systems and methods used in many studies. Here, indicate whether each material, system or method listed is relevant to your study. If you are not sure if a list item applies to your research, read the appropriate section before selecting a response.

Materials & experimental systems

n/a	Involved in the study	
☐	☒	Antibodies
☒	☐	Eukaryotic cell lines
☒	☐	Palaeontology and archaeology
☐	☒	Animals and other organisms
☒	☐	Clinical data
☒	☐	Dual use research of concern

Methods

n/a	Involved in the study	
☒	☐	ChIP-seq
☒	☐	Flow cytometry
☒	☐	MRI-based neuroimaging

Antibodies

Antibodies used	All of the antibody information is fully available in a table format in the Methods section Table 1.

Validation

Most of the commercial antibodies were validated by the suppliers and have been previously shown to work in the same types of experiments as indicated in Methods section Table 1. No further validation was performed in our hands.

Animals and other research organisms

Policy information about studies involving animals; ARRIVE guidelines recommended for reporting animal research, and Sex and Gender in Research

Laboratory animals

For the primary culture experiments at EPFL, only postnatal day 0 (P0) from WT C57BL/6JRj or a-Syn KO (C57BL/6J OlaHsd) mice strains were used. For primary culture experiments at University of Pennsylvania, timed pregnant CD1 mice were obtained as “adults” from the vendor - the embryos (E16-18) were used for studies. For PFF injections, B6C3F1/J mice were 2-3 months of age at the time of injection.

The ambient temperature at EPFL is maintained at 21°C (+/- 2°C) with humidity levels at 55% (+/- 10%) to ensure optimal health and well-being for the mice. The ambient conditions at University of Pennsylvania are 68-79°F and 30-70% humidity.

Animals were maintained on a 12-hour light/dark schedule and provided with food ad libitum.

Wild animals

No wild animals were used in this study.

Reporting on sex

Primary Neurons were collected from both sexes. Injected mice were all female, which is consistent with our previous injection experiments and allows for better direct comparisons with previous results with other fibril preparations.

Field-collected samples

No field-collected samples were used in this study.

Ethics oversight

Housing and procedures at University of Pennsylvania were performed according to the National Institutes of Health Guide for the Care and Use of Experimental Animals as approved by the University of Pennsylvania Institutional Animal Care and Use Committee. Housing and procedures at with École Polytechnique Fédérale de Lausanne were approved by the Swiss Federal Veterinary Office (authorization numbers VD3496).

Note that full information on the approval of the study protocol must also be provided in the manuscript.
