# Supplementary material for: O-GlcNAc forces an α-synuclein amyloid strain with notably diminished seeding and pathology
Source: Nat Chem Biol. 2024 Feb 12;20(5):646–55. doi: 10.1038/s41589-024-01551-2 (PMC11062923; doi:10.1038/s41589-024-01551-2)

Figure 2b

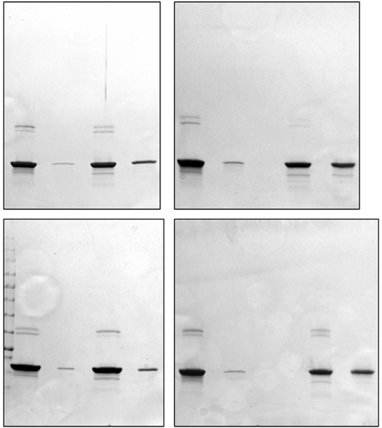

Figure 2e

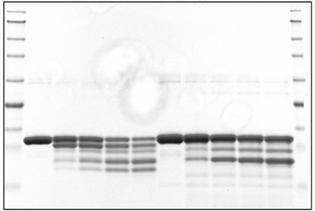

Figure 2f
(See Supplementary Figure 1)

Figure 2h
(See Supplementary Figure 2)

Figure 2g

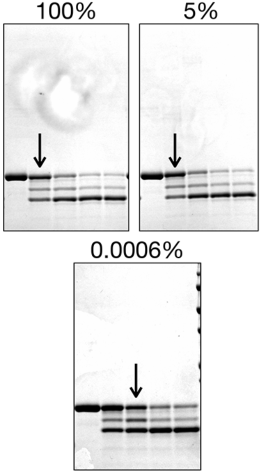

Figure 2i

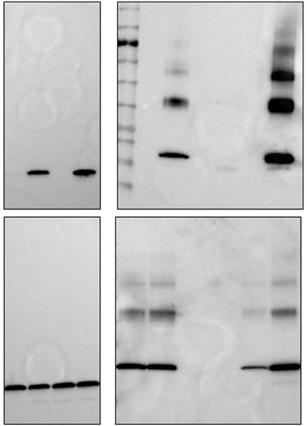

Supplement: Supplementary file 3 [file 41589_2024_1551_MOESM3_ESM.pdf]
